# Can we trust this short scale? Validation of a Brief Self-Report Measure of Epistemic Trust, Mistrust and Credulity (ETMCQ-R-6) in a sample of the U.K. general population

**DOI:** 10.1371/journal.pmen.0000591

**Published:** 2026-08-31

**Authors:** David Riedl, Henry Delamain, Peter Fonagy, Astrid Lampe, Samuel Eke, Tobias Nolte, Hanna Kampling, Johannes Kruse, Chloe Campbell

**Affiliations:** 1 Ludwig Boltzmann Institute for Rehabilitation Research, Vienna, Austria; 2 University Hospital of Psychiatry II, Department of Psychiatry, Psychotherapy, Psychosomatics and Medical Psychology, Medical University of Innsbruck, Innsbruck, Austria; 3 Research Department of Clinical, Educational and Health Psychology, University College London, London, United Kingdom; 4 CORE Data Lab, Centre for Outcomes Research and Effectiveness (CORE), Research Department of Clinical, Educational and Health Psychology, University College London, London, United Kingdom; 5 Anna Freud, London, United Kingdom; 6 VITREA Rehabilitation Center, Schruns, Austria; 7 Department of Psychosomatic Medicine and Psychotherapy, Justus Liebig University Giessen, Giessen, Germany; 8 Medical Center of the Philipps University Marburg, Marburg, Germany; University of Messina, ITALY

## Abstract

The concept of the epistemic stance (i.e., an individual’s openness to learning from others) has been increasingly recognized as a key construct associated with mental health. This study validated a six-item short form of the Epistemic Trust, Mistrust and Credulity Questionnaire (ETMCQ-R) in a UK quota-balanced community sample (N = 515). Using stepwise item reduction and confirmatory factor analysis, a three-factor structure with two items per subscale (trust, mistrust, credulity) was identified. The ETMCQ-R-6 showed acceptable fit (scaled *χ²*(6)=22.13, *p* = .001; robust CFI = .978; robust TLI = .945; robust RMSEA = .073 [90% CI:  .042,  .107]; SRMR = .029) and acceptable reliability for the trust (α = .75) and credulity (α = .77) subscales, while the mistrust subscale fell below the conventional threshold (α = ρ = ω = .68). Configural, metric, scalar, and structural invariance across gender and age groups was supported, while strict invariance was not established. Correlations between the long and short form were high (r = 0.83–0.87; φ = 0.95–1.00). Hierarchical regression analyses confirmed significant incremental predictive validity for epistemic mistrust and credulity across psychopathology outcomes, while the trust subscale demonstrated domain-specific incremental validity for attachment avoidance. The ETMCQ-R-6 offers a brief, psychometrically stable assessment of epistemic stance suitable for epidemiological research.

## 1 Background

The concept of the epistemic stance to better understand the etiology and development of psychopathology has increasingly gained attention over the last decade. The epistemic stance refers to an individual’s orientation toward social knowledge, encompassing their openness to learning from others and their positioning as both ‘providers of knowledge’ and ‘receivers of knowledge’ [1]. Its development is closely tied to early attachment security and personality organization in infancy, which together shape later capacities for social learning [2–4]. When this process unfolds typically, it supports the emergence of epistemic trust – the readiness to treat information from others as reliable, personally relevant, and generalizable [4]. Conversely, experiences of abuse, neglect, or insecure attachment can disrupt this trajectory, resulting in a tendency to view others’ communications as untrustworthy or malevolent and to resist influence. Alternatively, some individuals may show insufficient caution, accepting information indiscriminately and thus becoming susceptible to misinformation or exploitation. In the framework of the epistemic stance, these two forms of epistemic disruption are defined as epistemic mistrust and epistemic credulity [4,5]. It has been suggested that both mistrust and credulity, and the associated disturbances in social learning processes, may play a substantial role in the development and characterization of many types of psychopathology [3]. To facilitate empirical research in this field, the Epistemic Trust, Mistrust and Credulity Questionnaire (ETMCQ) [5] has been developed to evaluate the three dimensions of epistemic trust, mistrust and credulity based on structured individuals self-reports. The ETMCQ has since been adapted and validated in French [6], German [7], Italian [8], Persian [9], Serbian [10] and Argentine Spanish [11] and a large and constantly growing body of empirical evidence in line with the theoretical assumptions regarding the role of the epistemic stance has been produced. Recent empirical research has shown for example that epistemic trust is associated with post-traumatic stress disorder (PTSD) and complex post-traumatic stress disorder [12], borderline personality disorder [13], parental burn out [14], somatic symptom disorder [15], conspiracy endorsement [16], as well as physical diseases such as diabetes [17] and myocardial infarction [18]. In addition, observational studies have identified reductions in epistemic disruption to be associated with short [19] and long-term outcomes of psychodynamic treatment of cPTSD [20] as well as improvements during psychosomatic inpatient treatments [21].

Despite these generally positive empirical developments, psychometric evaluations of the ETMCQ have revealed three methodological difficulties. Firstly, the trust scale has shown substantially weaker correlations with outcomes compared to the mistrust and credulity scale across all available studies, which has led some researchers to question whether this scale should be removed from the questionnaire [22]. Secondly, item factor loadings were poor for some items and in one study some items even loaded onto an unexpected factor [8]. Thirdly, the internal consistencies of the subscales were found to be marginally or insufficient in some validation studies [8]. To account for these shortcomings, a revised version of the ETMCQ, namely the ETMCQ-R has been developed and validated [23]. The results of the validation study showed improved factorial validity, better internal consistencies and good external validity for all subscales.

Despite the good psychometric properties and theoretical coherence of the ETMCQ-R, its length with 18 items may limit its feasibility in large-scale surveys, clinical settings, or longitudinal studies where assessment time is constrained. One way to reduce participant burden, increasing completion rates and enabling the inclusion of epistemic stance assessment in multi-measure batteries, is the creation of an ETMCQ-R short form. This would further facilitate the integration of epistemic trust research into applied contexts such as clinical screening, epidemiological studies, and treatment monitoring, without substantial loss of psychometric precision.

The aim of this study was to develop and evaluate a brief six-item self-report measure derived from the ETMCQ-R and to examine whether the shortened scale preserves the factorial structure, reliability, and external validity patterns of the full instrument. We specifically aimed to test the factorial validity, internal consistency, and external validity of the derived short form as well as the comparability of the short and long form. To evaluate the construct validity of the ETMCQ-R-6, a theoretically motivated set of external criteria were included: attachment style was included since epistemic trust is theoretically grounded in early attachment security, with insecure attachment (particularly anxiety and avoidance) associated with epistemic hypervigilance or defensive closure toward interpersonal communication [3,4]. Mentalizing capacity was included since epistemic trust is conceptualized as a key facilitator of reflective functioning as it is assumed that individuals with greater certainty about their own and others’ mental states are better positioned for open social communication, while impaired mentalizing is linked to epistemic disruption [24]. Borderline personality features were included as a clinically relevant criterion since epistemic disruptions are central to the mentalization-based model of borderline pathology, and prior research has consistently shown stronger associations between epistemic disruption and borderline features than with general psychopathology [5,25]. General psychological distress was included as a broad indicator of psychopathology in line with the established association to epistemic disruption across multiple studies [3,12]. Perceived social support and resilience were included to reflect protective factors since epistemic trust is hypothesized to support adaptive social learning and coping, while mistrust and credulity were expected to show negative associations with both [4]. In sum, these criteria represent major facets of the nomological network of epistemic stance and allow evaluation whether the ETMCQ-R-6 preserves the construct validity of the full ETMCQ-R across a theoretically coherent range of outcomes.

## 2 Methods

### 2.1 Sample and procedure

The ETMCQ-R was administered between 16/03/2023 and 23/03/2023–525 adults recruited via the Prolific platform (https://www.prolific.com) to obtain a UK quota-balanced community sample. Demographic quotas ensured alignment with national distributions for age, gender, and ethnicity. All participants were UK residents aged 18 or older and fluent in English. Collected demographic data included age group (18–29, 30–39, 40–49, 50–59, ≥ 60 years), household income (<£30,000, ≥ £30,000, or prefer not to say), education level (secondary, university, postgraduate, none, or prefer not to say), ethnicity (White, minoritized group, or prefer not to say), gender (female, male, non-binary, or prefer not to say), and relationship status (married/in a relationship, single/widowed/divorced, or prefer not to say). Gender was assessed via Prolific’s standard demographic question, which asks participants to report their self-identified gender identity. For measurement invariance analyses, only participants identifying as female (n = 266) or male (n = 240) were included due to insufficient sample sizes in the non-binary (n = 5) and prefer not to answer (n = 4) categories to support multigroup confirmatory factor analysis (CFA) estimation.

### 2.2 Ethics statement

Written informed consent was obtained from all participants. The study adhered to the ethical standards of the Declaration of Helsinki (2013 revision) and received approval from the University College London Research Ethics Committee (ethics number: 20129/001).

### 2.3 Assessment instruments

The ETMCQ-R [23] is the revised version of the Epistemic Trust, Mistrust and Credulity Questionnaire (ETMCQ) [5] which was initially validated in this dataset. The ETMCQ-R includes 18 items which cover three dimensions of the individual’s epistemic stance: epistemic trust, epistemic mistrust and epistemic credulity. Good factorial and conceptual validity were observed for the ETMCQ-R [23].

The Experiences in Close Relationships–Revised (ECR-R) [26] is a 36-item self-report questionnaire that assesses adult attachment style. It employs a 7-point Likert scale (1 = strongly disagree to 7 = strongly agree) and generates two separate subscales for attachment anxiety and attachment avoidance with higher scores indicating more attachment anxiety or avoidance.

The Brief Symptom Inventory (BSI) [27] is a 18-item self-report measure designed to evaluate psychological distress. Responses are recorded on a 5-point Likert scale (0 = not at all to 4 = extremely). It covers three symptom dimensions, namely somatization, depression and anxiety with higher scores indicating higher symptom load.

The Personality Assessment Inventory–Borderline Scale (PAI-BOR) [28] consists of 24 items assessing four core features of borderline personality disorder: affective instability, identity disturbance, problematic relationships, and self-harm. Items are rated on a 4-point Likert scale (0 = false to 3 = very true), with six items per subscale. Higher scores indicate higher symptom load.

The Brief Resilience Scale (BRS) [29] measures perceived resilience through six items. Participants respond on a 5-point Likert scale (1 = strongly disagree to 5 = strongly agree), with both positively and negatively worded items included.

The Epistemic Vice Scale [30] is a newer 10-item self-report tool developed to assess dispositions that hinder knowledge acquisition and sharing. It is composed of two subscales: indifference and rigidity, each capturing distinct aspects of epistemic vice.

The Multidimensional Scale of Perceived Social Support (MSPSS) [31] is a 12-item measure of perceived support from family, friends, and significant others. Items are rated on a 7-point Likert scale (1 = very strongly disagree to 7 = very strongly agree), with four items per subscale. The Certainty About Mental States Questionnaire (CAMSQ) [32] assesses individual differences in people’s certainty about their own and others’ mental states as an aspect of the individuals mentalizing capabilities with 20 items on a 7-point Likert scale. The items can be divided in two 10 items subscales, namely self-certainty and other-certainty with higher scores indicating more certainty. In addition, a discrepancy score (Other-Certainty minus Self-Certainty) can be computed to indicate potential over- or under confidence in understanding others relative to oneself.

The Mentalization Questionnaire (MZQ) [33,34] assesses mentalizing impairments with 15 items on a 5-point Likert scale. The items in this analysis were reversely coded therefore a higher MZQ score indicates better mentalizing.

### 2.4 Statistics

We report how we determined our sample size, all data exclusions, all manipulations, and all measures in the study. To identify the optimal items for the ETMCQ-R-6 short form, a confirmatory factor analysis was calculated for the 18-item full version of the ETMCQ-R using the robust Maximum Likelihood estimator (MLR) with Yuan-Bentler scaling correction [35]. Items with lowest loadings on the specific factor were disregarded step by step to identify the items with highest factor loadings. Generally, a factor loading of *λ* ≥ 0.50 was deemed acceptable but we ideally expected loadings to be *λ* ≥ 0.60 – 0.70. If items showed a similar loading, internal consistencies were calculated (*ω* and *α*) to determine which item showed a higher consistency with the other items in the same scale. Finally, all potential items were evaluated based on their content coverage by the authors of the study. For each factor, two items which represented complementary facets of the underlying construct were chosen to avoid redundant wording or content and were assessed against the theoretical definitions of epistemic trust, mistrust, and credulity as specified in the foundational theoretical framework [4]. This ensured that the short form preserved the conceptual range of epistemic trust, mistrust, and credulity despite the substantial item reduction. For the *Trust subscale*, the included items were chosen to reflect benevolence appraisal (i.e., perceiving others’ communications as well-intentioned) and openness to the possible personal relevance of socially transmitted information. For the *Mistrust subscale*, the included items were chosen to reflect sensitivity to potential malevolence and protective resistance to influence. For the *Credulity subscale*, the included items were chosen to reflect suggestibility and over-openness to social information without appropriate discrimination. Consensus was reached through discussion among co-authors with expertise in epistemic trust theory, psychometric methodology, and clinical application, with final item assignments confirmed by the lead and senior authors. Thus, the final six items included in the ETMCQ-R-6 were chosen based on a joint criterion, based on item loading, internal consistency of the subscale and proximity to subscale content. We acknowledge that stepwise CFA-based item deletion does not represent the current state of the art for short-form development. Modern approaches such as Ant Colony Optimization or Genetic Algorithms allow simultaneous optimization of multiple psychometric criteria [36]. The current approach was selected for its interpretability and alignment with the derivation procedure of the parent ETMCQ-R. The limitations of this approach are discussed below.

All CFA and measurement invariance models were estimated using the robust Maximum Likelihood estimator (MLR) with Yuan-Bentler scaling correction, which provides scaled test statistics and robust standard errors appropriate for ordinal Likert-scale data [35]. To determine the factor solution’s goodness of fit, Pearson’s chi-squared test (*χ*^*2*^), Degree of Freedom (df), Tucker-Lewis Index (TLI), Comparative Fit Index (CFI), Standardized Root Mean Square Residual (SRMR), and Root Mean Square Error of Approximation (RMSEA) with lower and higher bounds of the 95% confidence interval (CI) were calculated. Acceptable goodness of fit was defined as RMSEA and SRMR values of <0.06-0.08 and CFI/TLI values >0.90 [37,38]. Factor loadings >0.40 were considered acceptable [39]. To evaluate potential overfitting of the reduced six-item model, the full dataset was randomly split into a 70% training subsample used for item selection and model estimation, and a 30% holdout subsample used for internal replication. The 70/30 ratio was selected to maximize the size of the derivation subsample while retaining an adequate holdout for comparison since a 50/50 split would have yielded a derivation sample of approximately n = 260, which would be insufficient for stable CFA estimation. Cases were allocated using simple random assignment with a fixed random seed. The proposed three-factor CFA was re-estimated in the holdout sample to assess the stability of model fit and factor loadings compared to the training sample.

To test measurement invariance of the ETMCQ-R-6, multigroup confirmatory factor was calculated for gender (male vs. female) and age groups. A sequence of increasingly constrained models was tested across groups. The constraints applied cumulatively across steps: in the first step the factor structure was freely estimated in each group to estimate configural invariance. In a second step, factor loadings were constrained equal to test for metric invariance. In a third step, item intercepts were constrained equal across groups to test scalar invariance. In a fourth step, residual variances were additionally constrained to test strict invariance. Before proceeding to multigroup models, the CFA model was estimated separately within each group to confirm adequate baseline fit. Invariance was evaluated using *χ²*-difference tests and changes in CFI (ΔCFI ≤ .010) [40] and RMSEA (ΔRMSEA < .015). Because the trust factor was represented by only two indicators and the initial multigroup specification showed signs of local estimation instability, a sensitivity analysis was additionally conducted in which the two trust-item loadings were constrained to equality. This alternative specification was used to examine whether the conclusions regarding gender invariance were robust to a more restrictive and numerically stable parameterization of the trust factor. Measurement Invariance Analyses are shown in S4 Table.

The internal consistencies of the two-item scales were evaluated using Cronbach’s α, with α ≥ 0.70 deemed acceptable, α ≥ 0.80 good and α ≥ 0.90 excellent. For two-item scales, the Spearman-Brown coefficient (ρ) is the most appropriate reliability index [41]: the Spearman-Brown Coefficient (*ρ*) and the inter-item correlations (*r*) were calculated additionally [41] with values of *r* ≥ 0.60 and *ρ* ≥ 0.70 deemed acceptable and *r* ≥ 0.70 and *ρ* ≥ 0.80 good. In addition, McDonald’s *ω* was computed to provide a model-based reliability index that does not assume tau-equivalence. For two-item scales, *ω* simplifies to the Spearman–Brown formula (*ω* = 2*r* / [1 + *r*]), and thus yields values equivalent to *ρ*. To complement these reliability estimates, the latent construct reliability (*H*) was calculated from the standardized factor loadings and residuals obtained in the CFA, with values of *H* ≥ 0.70 deemed acceptable [42]. The similarity to the ETMCQ-R was established using Pearson correlation coefficients with 95% CI, with values of *r* ≥ 0.80 deemed acceptable and *r* ≥ 0.90 as good [43]. In addition, Tucker’s congruence coefficient (*φ*) was calculated, with values of *φ* < 0.85 deemed unacceptable, *φ* = 0.85–0.94 acceptable to good and *φ* ≥ 0.95 as excellent [44].

To evaluate the construct validity of the ETMCQ-R-6 Pearson correlation coefficients were calculated between the long and short form of the ETMCQ-R with the BSI subscales, MZQ total score, CAMSQ subscales, ECR-R subscales, MSPSS subscales and BRS. The selection of external validity measures was informed by previous research as described in the introduction. In line with prior research and theoretical predictions, epistemic mistrust and credulity were expected to show positive associations with attachment insecurity, psychological distress, and borderline personality features, and negative associations with mentalizing capacity, perceived social support, and resilience. Epistemic trust was expected to show the opposite pattern across these domains, with the exception of borderline features and general psychopathology, for which weak or absent associations were anticipated based on prior findings [5,23]. Cohens *q* was calculated as an estimation of the effect size between correlations, with values of *q* < 0.10 deemed negligible, *q* = 0.1 – 0.3 as small, *q* = 0.3 – 0.5 as moderate and *q* > 0.5 as large [45]. The proportion of change in correlation magnitude is additionally reported in the tables as descriptive information.

To further examine the incremental predictive validity of the ETMCQ-R-6 subscales, hierarchical linear regression analyses were conducted. Given theoretical and empirical evidence linking epistemic mistrust most consistently to psychopathology, mistrust was entered in Step 1, followed by credulity (Step 2) and trust (Step 3). This approach allowed testing whether credulity and trust explain variance beyond an established epistemic risk-related stance. Since the ETMCQ-R-6 subscales and in particular mistrust and credulity share variance, the model was repeated with reversed order of entry as a sensitivity analysis. Mean change of explained variance (*ΔR^2^*) was interpreted as indicator of incremental predictive validity. Analyses were conducted with IBM SPSS (v30) and R (version 4.4.2; R Core Team, 2024) running in RStudio (version 2026.01.1 Build 403; Posit Software, 2026). All CFA analyses were done using the lavaan package (version 0.6-21) [46]. *P*-values below 0.05 were considered statistically significant.

## 3 Results

### 3.1 Sample description

Of the initially n = 525 participants, n = 10 did not complete the ETMCQ-R and were therefore excluded from the analysis. The remaining n = 515 (98.1%) were included in the analysis. The mean age in the included sample was 44.8 years and a slight majority was female (51.7%). The largest proportion of participants reported a low annual income (37.1%) and the majority of the sample was married or in a relationship (65.2%). For details, see Table 1.

**Table 1:** Demographic data.

		n	%
Age groups	< 30 years	113	21.9%
(mean age: 44.8 + /- 16.0 years)	30-40 years	100	19.4%
	40-50 years	85	16.5%
	50-60 years	105	20.4%
	>60 years	112	21.7%
Annual household income	low income (<£ 30,000)	191	37.1%
	middle income (£ 30,000 - £ 50,000)	132	25.6%
	high income (> £ 50,000)	163	31.7%
	*missing*	*29*	*5.6%*
Educational level	low to mid education	185	35.9%
	high education	327	63.5%
	*missing*	*3*	*0.6%*
Gender	Female	266	51.7%
	Male	240	46.6%
	Non-Binary	5	1.0%
	*missing*	*4*	*0.8%*
Relationship status	single / widowed / divorced	176	34.2%
	married / in relationship	336	65.2%
	*missing*	*3*	*0.6%*

Low to mid education included no formal education, secondary school, A-levels, GCSEs, O-levels, CSEs while high education included a university degree or equivalent professional qualification (e.g., BA/BSc, NVQ level 4 or equivalent) or post graduate qualifications (e.g., MA/MSc, doctorate, MBA, NVQ level 5).

### 3.2 Item reduction

**Fig 1:**
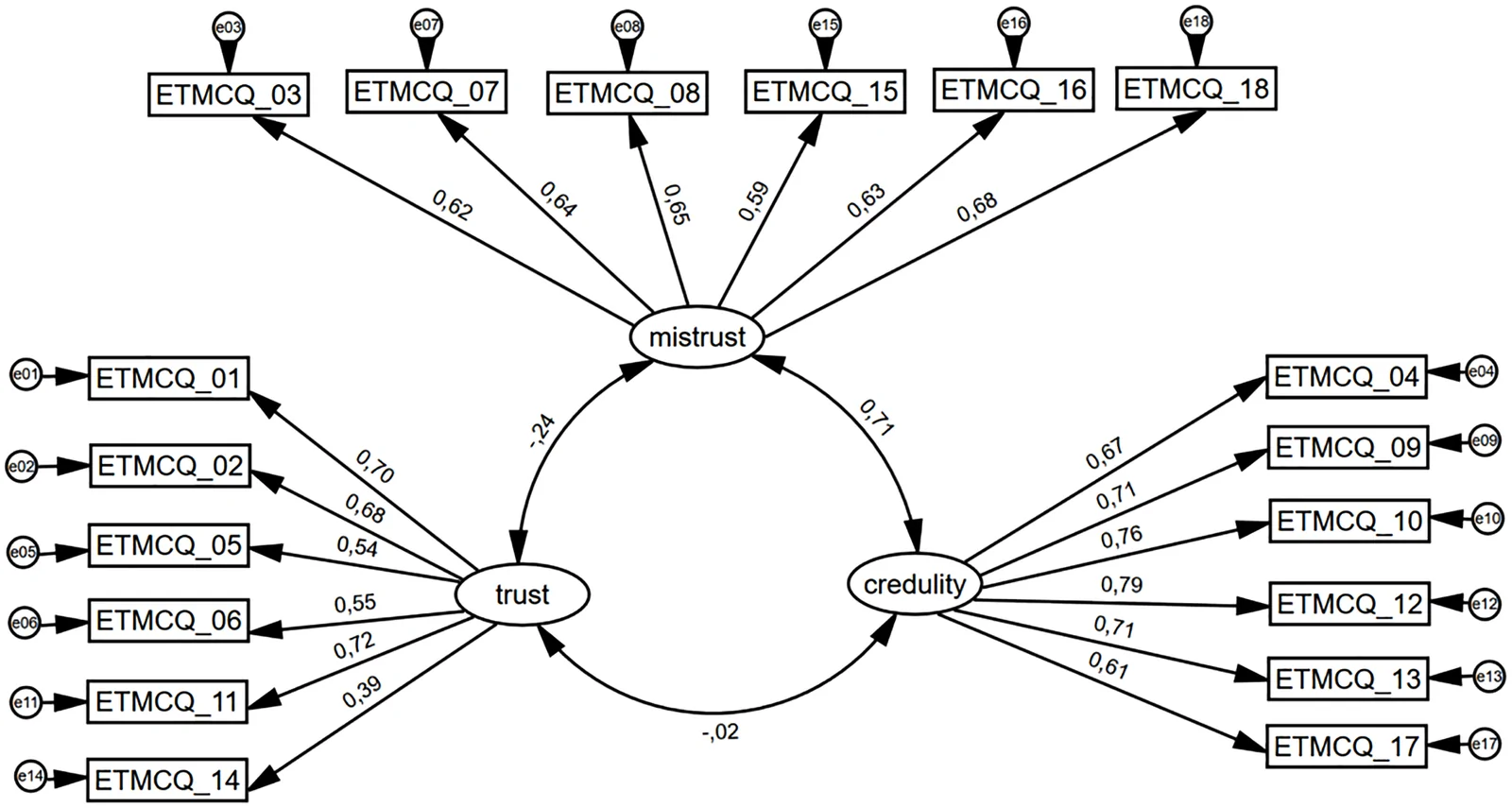
Confirmatory factor analysis for the Revised Epistemic Trust, Mistrust, Credulity Scale (ETMCQ-R). Rectangles represent items and circles represent error terms **(e)**. Numbers next to arrows in the model represent standardized estimates.

In a first step, a confirmatory factor analysis was calculated for the proposed three factor model of the ETMCQ-R as described previously [23] using the robust Maximum Likelihood estimator (MLR) with Yuan-Bentler scaling correction. The factor loadings ranged between 0.39 –0.72 for the trust factor with highest loadings for items 11 (*λ* = 0.72) and item 1 (*λ* = 0.70). For the mistrust factor, loadings ranged between 0.59 – 0.68 with highest loading for item 18 (*λ* = 0.68) followed, by item 8 (*λ* = 0.65) and item 7 (*λ* = 0.64). For the credulity factor, the item loadings ranged between 0.61 – 0.79 with highest loadings for item 12 (*λ* = 0.79) and item 9 (*λ* = 0.76). For details, see Fig 1.

The individual items with lowest factor loadings were then deleted step by step for each subscale. For the trust subscale, item 1 and 11 remained the highest loading items. For the mistrust subscale, the items 7 (*λ* = 0.64) and item 8 (*λ* = 0.65) showed slightly higher factor loadings than item 18 (*λ* = 0.68). However, the analysis of Cronbach Alpha showed a slightly higher internal consistency if item 18 was deleted. Items 7 and 8 were therefore retained on the joint criterion of loading strength and internal consistency. Similarly, for the credulity factor item 10 (*λ* = 0.76) and item 9 (*λ* = 0.71) showed a slightly lower factor loading than item 12 (*λ* = 0.79) but the analysis of internal consistency supported excluding item 12.

**Fig 2:**
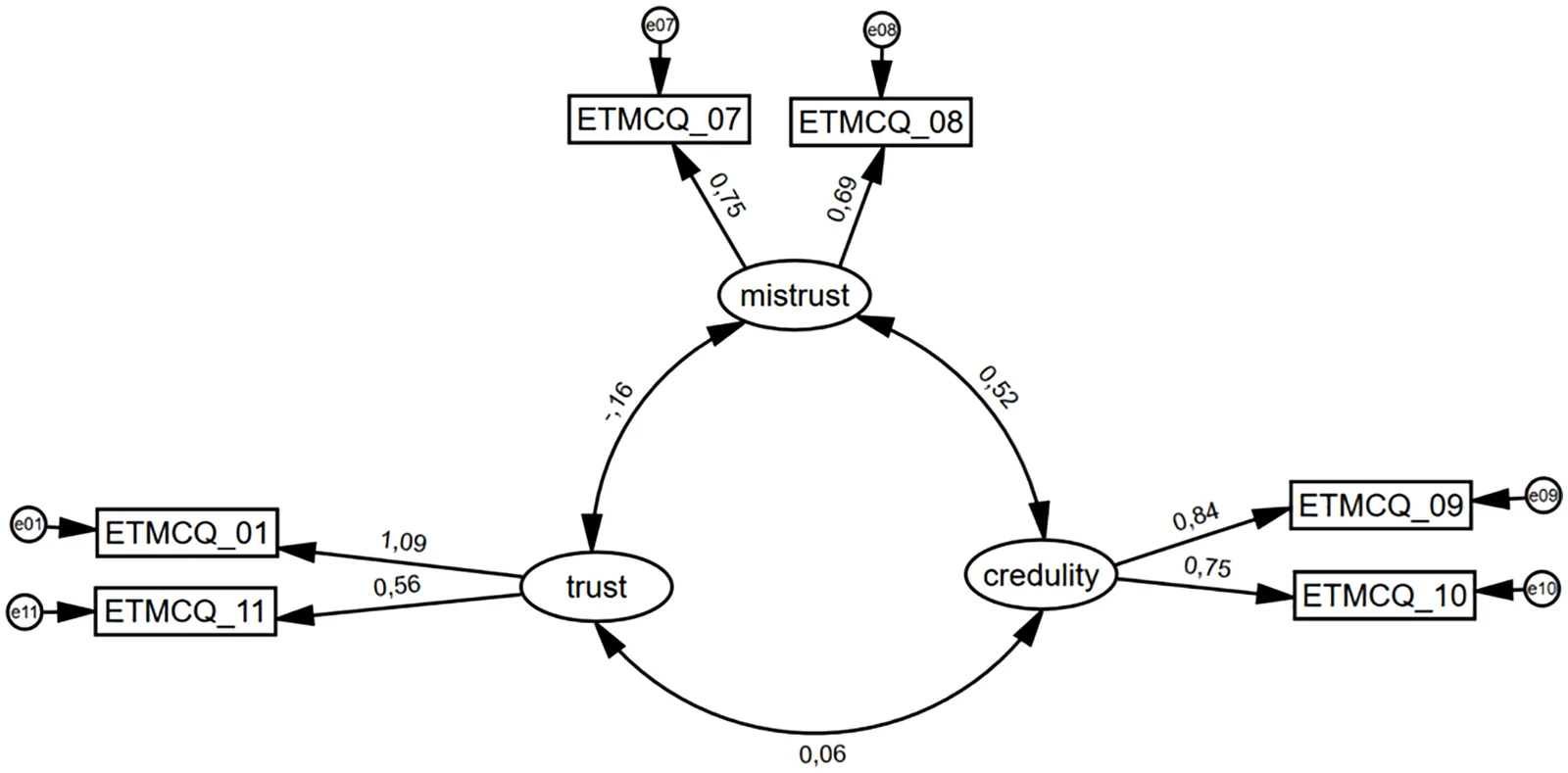
Confirmatory factor analysis for the six item short form of the Revised Epistemic Trust, Mistrust, Credulity Scale (ETMCQ-R-6). Rectangles represent items and circles represent error terms **(e)**. Numbers next to arrows in the model represent standardized estimates.Note. The standardized loading for Trust Item 1 exceeds 1.0 (λ = 1.085), constituting a Heywood case and an inadmissible parameter estimate. This reflects estimation instability, consistent with the known vulnerability of two-indicator factors to empirical under-identification in small to moderate samples [47,48]. See Discussion for details. All residual variances were constrained to be non-negative.

The final model for the ETMCQ-R-6 therefore consisted of items 1 and 11 for the trust subscale, items 7 and 8 for the mistrust subscale and items 9 and 10 for the credulity subscale. In our sample, acceptable fit indices were found for the final model (scaled *χ*^*2*^ = 22.13, *p* = 0.001; TLI = 0.945; CFI = 0.978; SRMR = 0.029; RMSEA = 0.073; 95% CI [0.042 – 0.107]; PCLOSE = 0.10). The standardized loading for trust item 1 (λ = 1.085) constituted a Heywood case and an inadmissible parameter estimate. Such solutions can arise in CFA as a form of estimation instability, particularly under conditions of sampling fluctuation or empirical under-identification in small to moderate samples. It has been argued that two-indicator factors may be especially vulnerable because they provide limited information for estimating the latent construct [47,48]. Residual variances were constrained to be non-negative. The implications of this finding and its resolution in an independent large sample are discussed below. For details, see Fig 2.

A 70/30 holdout validation was conducted to evaluate potential overfitting of the six-item ETMCQ-R model following item reduction. In the 70% training subsample (N = 358), the proposed three-factor CFA model demonstrated good fit under MLR estimation (scaled *χ²*(6) = 22.71, *p* = .001, CFI = .971, TLI = .927, RMSEA = .084, 95% CI [0.049 – 0.123]. The model was re-estimated in the 30% holdout subsample (N = 157), with acceptable and comparable model fit results (scaled *χ²*(6) = 13.06, *p* = .042, CFI = .972, TLI = .929, RMSEA = .086, 95% CI [0.015 – 0.151]). Standardized factor loadings remained statistically significant and in the expected range for mistrust (*λ* = 0.74 – 0.78) and credulity (*λ* = 0.60 – 1.00), thus indicating stable measurement properties across the two samples. For the trust subscale, the Heywood case observed in the full derivation sample was aggravated in both subsamples, consistent with the known vulnerability of two-indicator factors to empirical under-identification in small to moderate samples [47,48]. The trust subscale loadings are therefore not reported for the holdout validation. Their cross-sample resolution is addressed in the independent German replication reported below.

A table with mean scores and standard deviation for all items as well as and factor loadings, communalities and corrected item-total correlation for the 18-item and 6-item version are shown in S3 Table.

### 3.3 Independent replication in a German representative sample

To address the limitation of development and validation within the same sample, the proposed factorial structure of the ETMCQ-R-6 was additionally examined in an independent sample from a nationally representative German general population survey collected through random route sampling (N = 2,522; Riedl et al., under review). Specifically, a confirmatory factor analysis was conducted using MLR estimation (Riedl et al., under review). The hypothesized three-factor model demonstrated excellent fit (χ²(6) = 58.74, p < .001; CFI = .984; TLI = .961; RMSEA = .060 [90% CI:  .047,  .074]; SRMR = .069). All standardized factor loadings were statistically significant (p < .001) and fell within admissible bounds, ranging from λ = .57 to λ = .89 across the three subscales. No Heywood cases or inadmissible parameter estimates occurred in this sample. These findings underscore the validity of the proposed three-factor structure for the ETMCQ-R-6 and indicates that the psychometric properties observed in the UK sample are generalizable to the German general population.

### 3.4 Measurement invariance

Prior to multigroup confirmatory factor analyses, the ETMCQ-R-6 model was estimated separately within each gender group to confirm adequate baseline fit. The model demonstrated acceptable fit in females (robust CFI = .991, RMSEA = .048 [.000,  .101], SRMR = .031). In males, however, baseline fit fell below conventional thresholds (robust CFI = .944, RMSEA = .109 [.087,  .133], SRMR = .045), indicating that the three-factor model does not fit the male subsample adequately when freely estimated. Evaluation of parameter estimates showed that this misfit was localized to the Trust subscale, where the Heywood case observed in the full sample was concentrated in the male group. Given this baseline misfit, the gender invariance findings reported below should be interpreted with caution, and the results are best understood as reflecting the measurement properties of the Mistrust and Credulity subscales rather than the full six-item instrument. A sensitivity analysis using an equality constraint on the two Trust item loadings partially stabilized the model in males (robust CFI = .959, RMSEA = .086), though fit remained below conventional thresholds, further confirming that the Trust subscale is the source of gender-related measurement instability.

A series of multigroup CFA models were then estimated to evaluate gender invariance of the ETMCQ-R-6. The constraints applied cumulatively across steps. The configural model, in which factor structure was freely estimated across groups, showed acceptable fit (robust CFI = .971, RMSEA = .083 [.058,  .109]), indicating that the overall factor structure was comparable across men and women. Factor loadings were additionally constrained equal across groups to test metric invariance. This constraint did not significantly worsen model fit (Δχ²(3) = 0.57, p = .904, ΔCFI = .010, ΔRMSEA = .022), supporting metric invariance. Item intercepts were additionally constrained equal to test scalar invariance, again without significant deterioration (Δχ²(3) = 4.95, p = .176, ΔCFI = .003, ΔRMSEA = .002), supporting scalar invariance. Factor variances and covariances were then constrained equal to test structural invariance, with model fit remaining stable (Δχ²(6) = 3.25, p = .777, ΔCFI = .004, ΔRMSEA = .013), supporting structural invariance. Finally, residual variances were additionally constrained equal to test strict invariance. This resulted in a significant deterioration of model fit (Δχ²(6) = 17.06, p = .009, ΔCFI = .018, ΔRMSEA = .013), indicating that strict invariance was not supported. Taken together, the results support configural, metric, scalar, and structural invariance of the ETMCQ-R-6 across gender, while strict invariance could not be established.

As a sensitivity analysis, the gender invariance models were re-estimated using an alternative specification in which the two Trust item loadings were constrained to equality to stabilize the Trust factor. Under this specification, configural (robust CFI = .972), metric (Δχ²(1) = 0.72, p = .398, ΔCFI = .001), scalar (Δχ²(3) = 7.65, p = .054, ΔCFI = .007), and structural invariance (Δχ²(6) = 2.81, p = .832, ΔCFI = .006) were all supported, while strict invariance was not (Δχ²(6) = 16.26, p = .012, ΔCFI = .018). The substantive conclusions regarding the gender comparability of the ETMCQ-R-6 were therefore robust across model specifications.

A parallel sequence of multigroup CFA models was estimated to evaluate age invariance across five age groups (<30, 30–39, 40–49, 50–59, 60 + years). The configural model showed acceptable fit (robust CFI = .985, RMSEA = .060 [.000,  .098]). Metric invariance was supported, with factor loadings constrained equal across age groups without significant deterioration (Δχ²(12) = 12.41, p = .413, ΔCFI = .001, ΔRMSEA = .009). Scalar invariance was similarly supported, with item intercepts additionally constrained equal (Δχ²(12) = 14.15, p = .291, ΔCFI = .003, ΔRMSEA = .001). Structural invariance, with factor variances and covariances additionally constrained showed borderline acceptable results (Δχ²(24) = 33.25, p = .099, ΔCFI = .015, ΔRMSEA = .006), suggesting that latent factor relationships vary to some degree across age groups. Strict invariance was not supported (Δχ²(24) = 45.69, p = .005, ΔCFI = .034, ΔRMSEA = .013). Taken together, the results support configural, metric, and scalar invariance of the ETMCQ-R-6 across age groups, while structural and strict invariance could not be fully established.

Descriptive statistics and percentile-based cutoffs for the ETMCQ-R-6 subscales by age group and gender are presented in S2 Table.

### 3.5 Reliability and item-level properties of the ETMCQ-R-6

The subscales mistrust was negatively associated with trust (*r* = -.37, *p* < .001) and credulity (*r* = -.12, *p* = .008), while trust was not significantly associated with credulity (*r* = .08, *p* = .09). Each of the six items demonstrated adequate variability across the full 7-point response range [1–6], suggesting that the shortened measures captured a broad and meaningful range of responses. Participants reported moderate levels of trust (M = 4.52, SD = 1.39) and mistrust (M = 3.98, SD = 1.24), whereas generally lower levels were observed for credulity (M = 2.58, SD = 1.27), indicating that participants tended to report more trust than credulity. As for the distribution of responses, the trust subscale was slightly negatively skewed (-0.47) and showed low kurtosis (-0.42), suggesting a modest clustering toward higher scores without truncation at the upper end. Mistrust was nearly symmetric (skew = 0.08, kurtosis = -0.24), while credulity showed a moderate positive skew (0.93) and mild kurtosis (0.43), reflecting a somewhat greater concentration of lower scores, yet still a sufficient spread of responses. Acceptable internal consistencies (*α* = 0.75, *ρ* = 0.75, *ω* = .78) and inter-item correlations (*r* = .60; 95% CI [.55 –  .66]) for the trust scale as well as for the credulity scale (*α* = 0.77, *ρ* = 0.77; *ω* = 0.77; *r* = .63; 95% CI [.57 –  .68]). In addition, acceptable to good latent construct reliability was found for trust (*H* = 1.0) and credulity (*H* = 0.79). For the mistrust scale, all reliability indices fell below the conventional threshold (*α* = 0.68, *ρ* = 0.68; *ω* = 0.68; *r* = .51; 95% CI [.45 –  .57]). The convergence of all indices at the same value confirms that this represents a genuine reliability limitation rather than an underestimation artefact specific to Cronbach’s alpha. The latent construct reliability was similarly below the acceptable threshold (*H* = 0.68). The practical implications of this finding are discussed below. To evaluate the similarity between the ETMCQ-R and ETMCQ-R-6 subscales, correlation coefficients and Tucker’s congruence coefficient were calculated. For the trust scale an acceptable correlation was found between the full and short scale (*r* = .84; 95% CI [.81 –  .86]) excellent congruence was observed (*φ* = .95). Similarly, acceptable correlation and excellent congruence was found for mistrust (*r* = .83; 95% CI [.81 –  .86]; *φ* = 1.00) and credulity (*r* = .87; 95% CI [.84 –  .89]; *φ* = .99).

Factor loadings, communalities and item-total correlations for the ETMCQ-R and ETMCQ-R-6 items are shown in S3 Table.

### 3.6 Construct validity

To evaluate construct validity, the correlations of the ETMCQ-R-6 with external measures were compared to patterns of the ETMCQ-R. Differences of Cohens *q* < 0.10 were deemed negligible, *q* = 0.1 – 0.3 as small, *q* = 0.3 – 0.5 as moderate and *q* > 0.5 as large. For the ETMCQ-R-6 trust scale, correlations with external measures were highly similar to those of the full version, with negligible or small changes in magnitude across most variables (*Δr*  ≤ .04; *q* ≤ .04). Correlations with the CAMSQ subscales also remained consistent, both for Self-Certainty (*Δr* = -.08, *q* = .08) and Other-Certainty (*Δr* = -.06, *q* = .06), as well as Discrepancy (Δr = .03, q = .03). Overall, these differences fall within the acceptable range, indicating that the shortened trust subscale retains its external validity in relation to mentalization-related constructs. For details, see Table 2 and S1 Table.

**Table 2:** Evaluation of external validity for the ETMCQ-R-6 trust scale.

	ETMCQ-R		ETMCQ-R-6				
	r	95% CI	r	95% CI	Δr	% change	Cohen’s q
ECR-R Anxiety	-0.06	[-0.141, 0.032]	-0.09	[-0.177, -0.004]	-0.04	-65.5	0.04
ECR-R Avoidance	0.37	[0.296, 0.446]	0.35	[0.274, 0.427]	-0.02	-5.4	0.02
MZQ total	0.15	[0.062, 0.232]	0.18	[0.091, 0.260]	0.03	19.6	0.03
BSI Depression	-0.07	[-0.153, 0.020]	-0.1	[-0.189, -0.017]	-0.04	-55.2	0.04
BSI Anxiety	0.02	[-0.065, 0.109]	0.02	[-0.070, 0.104]	0.00	-22.7	0.01
BRS	0.06	[-0.025, 0.148]	0.08	[-0.002, 0.171]	0.02	37.1	0.02
MSPSS Total	0.41	[0.337, 0.482]	0.41	[0.334, 0.479]	0.00	-0.7	0.00
MSPSS Significant Other	0.24	[0.161, 0.325]	0.26	[0.179, 0.341]	0.02	6.9	0.02
MSPSS Family	0.32	[0.236, 0.393]	0.33	[0.246, 0.402]	0.01	2.8	0.01
MSPSS Friends	0.45	[0.377, 0.516]	0.41	[0.338, 0.483]	-0.04	-8.0	0.04
PAI-BOR	-0.04	[-0.122, 0.052]	-0.03	[-0.119, 0.055]	0.01	8.6	0.00
PAI-BOR: Affective Instability	-0.067	[-0.153, 0.020]	-0.064	[-0.151, 0.023]	0.003	-4.5	0.003
PAI-BOR: Identity Problems	-0.015	[-0.102, 0.072]	-0.014	[-0.101, 0.073]	0.001	-6.7	0.001
PAI-BOR: Negative Relationships	0.026	[-0.061, 0.113]	0.024	[-0.063, 0.111]	-0.002	-7.7	-0.002
PAI-BOR: Self-Harm / Impulsivity	-0.081	[-0.167, 0.006]	-0.068	[-0.154, 0.019]	0.013	-16	0.013
EVS Indifference	-0.102	[-0.187, -0.015]	0.013	[-0.074, 0.100]	0.115	-112.7	0.115
EVS Rigidity	-0.088	[-0.174, -0.002]	-0.006	[-0.093, 0.081]	0.082	-93.2	0.082
CAMSQ Self-Certainty	0.33	[0.249, 0.404]	0.25	[0.167, 0.330]	-0.08	-24.3	0.08
CAMSQ Other-Certainty	0.27	[0.189, 0.350]	0.21	[0.128, 0.294]	-0.06	-23.0	0.06
CAMSQ Discrepancy (Other – Self)	-0.09	[-0.176, -0.003]	-0.06	[-0.143, 0.030]	0.03	-33.3	0.03

CI = confidence interval; Δr = mean change between the correlation coefficient of the ETMCQ-R and the ETMCQ-R-6 trust scale with the external questionnaires; % change = Δr in percentage. Cohens q = effect size between correlations; q < 0.10 was defined as negligible, q = 0.1 – 0.3 as small, q = 0.3 – 0.5 as moderate and q > 0.5 as large. ECR-R = Experiences in Close Relationships-Revised; CAMSQ = Certainty About Mental States Questionnaire; MZQ = Mentalization Questionnaire; BSI = Brief Symptom Inventory (18 items); BRS = Brief Resilience Scale; MSPSS = Multidimensional Scale of Perceived Social Support; PAI-BOR = Personality Assessment Inventory-Borderline Scale.

For the ETMCQ-R-6 mistrust scale, correlations with external measures were highly similar to those of the full version. Across all variables, differences were small to moderate, with *Δr* values ranging from -.05 to -.12 (|*q*| ≤ .17). Associations with indicators of attachment anxiety (ECR-R Anxiety: *Δr* = -.11, *q* = .13) and borderline traits (PAI-BOR total: *Δr* = -.08, *q* = .11) remained robust, suggesting good retention of construct validity. Slightly larger proportional reductions were observed for measures of social support (e.g., MSPSS Family: *Δr* = .09, 33%; MSPSS Total: *Δr* = .09, 28%). Correlations with rigidity and indifference from the EVS decreased more noticeably (*Δr* = -.08 to -.09; 27 – 37%). Overall, the shortened mistrust scale preserved the core external validity profile of the full version, with only minor attenuation in strength for some interpersonal and attitudinal measures. For details, see Table 3.

**Table 3:** Evaluation of external validity for the ETMCQ-R-6 mistrust scale.

	ETMCQ-R		ETMCQ-R-6				
	r	95% CI	r	95% CI	Δr	% change	Cohen’s q
ECR-R Anxiety	0.45	[0.376, 0.515]	0.33	[0.254, 0.409]	-0.11	-25.4	0.13
ECR-R Avoidance	-0.13	[-0.219, -0.047]	-0.09	[-0.177, -0.004]	0.04	32.1	0.04
MZQ total	-0.60	[-0.656, -0.545]	-0.48	[-0.548, -0.414]	0.12	19.7	0.17
BSI Depression	0.39	[0.311, 0.460]	0.32	[0.244, 0.400]	-0.06	-16.5	0.07
BSI Anxiety	0.39	[0.313, 0.461]	0.31	[0.229, 0.387]	-0.08	-20.5	0.09
BRS Mean	0.23	[0.147, 0.312]	0.21	[0.124, 0.291]	-0.02	-9.5	0.02
MSPSS Total	-0.32	[-0.397, -0.241]	-0.23	[-0.311, -0.146]	0.09	28.3	0.10
MSPSS Significant Other	-0.18	[-0.266, -0.097]	-0.13	[-0.218, -0.046]	0.05	27.3	0.05
MSPSS Family	-0.27	[-0.353, -0.191]	-0.18	[-0.267, -0.098]	0.09	32.8	0.10
MSPSS Friends	-0.33	[-0.406, -0.250]	-0.25	[-0.328, -0.164]	0.08	24.8	0.09
PAI-BOR	0.54	[0.479, 0.602]	0.46	[0.390, 0.527]	-0.08	-15.1	0.11
PAI-BOR: Affective Instability	0.52	[0.452, 0.580]	0.44	[0.362, 0.503]	-0.084	-16.2	-0.109
PAI-BOR: Identity Problems	0.50	[0.431, 0.562]	0.42	[0.346, 0.489]	-0.079	-15.8	-0.1
PAI-BOR: Negative Relationships	0.47	[0.402, 0.537]	0.40	[0.324, 0.471]	-0.073	-15.4	-0.09
PAI-BOR: Self-Harm / Impulsivity	0.44	[0.372, 0.512]	0.39	[0.317, 0.464]	-0.051	-11.5	-0.062
EVS Indifference	0.28	[0.195, 0.356]	0.20	[0.117, 0.284]	-0.075	-27.1	-0.08
EVS Rigidity	0.23	[0.149, 0.314]	0.15	[0.061, 0.231]	-0.086	-36.9	-0.089
CAMSQ Self-Certainty	-0.17	[-0.249, -0.080]	-0.11	[-0.191, -0.019]	0.06	-34.2	0.06
CAMSQ Other-Certainty	-0.19	[-0.268, -0.100]	-0.13	[-0.213, -0.042]	0.06	-31.3	0.06
CAMSQ Discrepancy (Other – Self)	-0.05	[-0.132, 0.042]	-0.05	[-0.135, 0.039]	0.00	-6.4	0.00

CI = confidence interval; Δr = mean change between the correlation coefficient of the ETMCQ-R and the ETMCQ-R-6 mistrust scale with the external questionnaires; % change = Δr in percentage. Cohens q = effect size between correlations; q < 0.10 was defined as negligible, q = 0.1 – 0.3 as small, q = 0.3 – 0.5 as moderate and q > 0.5 as large.

For the ETMCQ-R-6 credulity subscale, reductions in correlation compared to the full version were found with *Δr* values ranging from -.04 to -.14 (|*q*| ≤ .20). Associations with key indicators of mentalization and borderline pathology (e.g., MZQ total and PAI-BOR subscales) remained strong, suggesting that the short form adequately captures the core construct. Slight attenuation was observed for measures of social support (e.g., MSPSS Friends, *Δr* = .11, *q* = .11) and attachment anxiety (*Δr* = -.09, *q* = .11).Correlations with the CAMSQ subscales (Self-Certainty, Other-Certainty, Discrepancy) (*Δr* = .01–.06; *q* ≤ .06) indicated consistent relationships with certainty about mental states despite item reduction. Overall, the ETMCQ-R-6 credulity scale retained good external validity, with only minor information loss across domains related to interpersonal trust and metacognitive certainty. For details, see Table 4.

**Table 4:** Evaluation of external validity for the ETMCQ-R-6 credulity scale.

	ETMCQ-R		ETMCQ-R-6				
	r	95% CI	r	95% CI	Δr	% change	Cohen’s q
ECR-R Anxiety	0.48	[0.409, 0.543]	0.39	[0.313, 0.460]	-0.09	-18.8	0.11
ECR-R Avoidance	-0.14	[-0.220, -0.050]	-0.07	[-0.156, 0.017]	0.07	48.5	0.07
MZQ total	-0.58	[-0.636, -0.520]	-0.44	[-0.504, -0.363]	0.14	25.0	0.20
BSI Depression	0.37	[0.291, 0.442]	0.26	[0.182, 0.344]	-0.10	-28.2	0.12
BSI Anxiety	0.38	[0.306, 0.455]	0.30	[0.213, 0.373]	-0.09	-23.0	0.10
BRS Mean	0.25	[0.169, 0.332]	0.22	[0.141, 0.306]	-0.03	-10.7	0.03
MSPSS Total	-0.23	[-0.313, -0.148]	-0.12	[-0.206, -0.034]	0.11	47.8	0.11
MSPSS Significant Other	-0.17	[-0.250, -0.080]	-0.10	[-0.183, -0.010]	0.07	41.6	0.07
MSPSS Family	-0.21	[-0.288, -0.121]	-0.11	[-0.198, -0.026]	0.09	45.1	0.10
MSPSS Friends	-0.19	[-0.276, -0.109]	-0.08	[-0.170, 0.003]	0.11	56.7	0.11
PAI-BOR	0.55	[0.483, 0.605]	0.48	[0.411, 0.545]	-0.07	-12.1	0.09
PAI-BOR: Affective Instability	0.52	[0.453, 0.580]	0.457	[0.385, 0.523]	-0.063	-12.1	-0.08
PAI-BOR: Identity Problems	0.493	[0.424, 0.556]	0.426	[0.352, 0.495]	-0.067	-13.6	-0.09
PAI-BOR: Negative Relationships	0.504	[0.437, 0.567]	0.443	[0.370, 0.510]	-0.061	-12.1	-0.08
PAI-BOR: Self-Harm / Impulsivity	0.427	[0.353, 0.495]	0.387	[0.310, 0.459]	-0.04	-9.4	-0.05
EVS Indifference	0.31	[0.229, 0.386]	0.257	[0.174, 0.336]	-0.053	-17.1	-0.06
EVS Rigidity	0.19	[0.105, 0.273]	0.189	[0.104, 0.272]	-0.001	-0.5	-0.001
CAMSQ Self-Certainty	-0.25	[-0.329, -0.166]	-0.19	[-0.274, -0.106]	0.06	-23.2	0.06
CAMSQ Other-Certainty	-0.31	[-0.386, -0.228]	-0.25	[-0.327, -0.164]	0.06	-19.4	0.06
CAMSQ Discrepancy (Other – Self)	-0.13	[-0.212, -0.040]	-0.12	[-0.201, -0.029]	0.01	-8.7	0.01

CI = confidence interval; Δr = mean change between the correlation coefficient of the ETMCQ-R and the ETMCQ-R-6 credulity scale with the external questionnaires; % change = Δr in percentage. Cohens q = effect size between correlations; q < 0.10 was defined as negligible, q = 0.1 – 0.3 as small, q = 0.3 – 0.5 as moderate and q > 0.5 as large.

### 3.7 Incremental predictive validity of the ETMCQ-R-6 subscales

Hierarchical regression analyses were conducted to examine whether each ETMCQ-R-6 subscale contributed unique variance to general psychological distress and borderline personality features beyond the other subscales, adjusting for gender, age, income, education and marital status.

For general psychological distress (BSI Global Severity Index), mistrust explained significant variance in Step 1 (adjusted R^2^ = .152, λ = .299, p < .001). Credulity accounted for additional unique variance in Step 2 (ΔR^2^ = .019, adjusted R^2^ = .169, λ = .152, p = .001), indicating incremental validity beyond mistrust. Trust did not contribute significant additional variance in Step 3 (ΔR^2^ = .005, adjusted R^2^ = .172, λ = -.074, p = .098), suggesting that trust does not independently predict general psychological distress once mistrust and credulity are accounted for. The three ETMCQ-R-6 subscales explained 18.7% of variance in general psychological distress (adjusted R^2^ = .172). Sensitivity analyses with reversed entry order confirmed the robustness of these findings: when trust was entered first (adjusted R^2^ = .075, p = .027), credulity (ΔR^2^ = .057, λ = .247, p < .001) and mistrust (ΔR^2^ = .043, λ = .230, p < .001) each retained significant incremental contributions regardless of entry order.

For borderline personality features (PAI-BOR total score), mistrust explained substantial variance in Step 1 (adjusted R^2^ = .288, λ = .436, p < .001). Credulity contributed significant additional variance in Step 2 (ΔR^2^ = .069, adjusted R^2^ = .357, λ = .290, p < .001), demonstrating incremental validity beyond mistrust. Trust did not contribute significant additional variance in Step 3 (ΔR^2^ = .004, adjusted R^2^ = .359, λ = -.065, p = .101). The three ETMCQ-R-6 subscales together with covariates explained 37.0% of variance in borderline personality features (adjusted R^2^ = .359). Sensitivity analyses showed that trust significantly explained variance when entered first (adjusted R^2^ = .113, p = .026) and both credulity (ΔR^2^ = .163, λ = .417, p < .001) and mistrust (ΔR^2^ = .082, λ = .320, p < .001) retained strong incremental contributions regardless of entry order.

For attachment anxiety, mistrust explained significant variance in Step 1 (adj. R^2^ = .192, p < .001). Credulity contributed additional incremental variance in Step 2 (Δadj. R^2^ = .066, β = .293, p < .001), and trust contributed a small but significant further increment in Step 3 (Δadj. R^2^ = .005, β = -.083, p = .049). The three subscales and covariates explained 26.2% of variance in attachment anxiety. Sensitivity analyses confirmed that mistrust and credulity each retained significant incremental contributions regardless of entry order, with trust additionally reaching significance in the final model across all entry orders.

For attachment avoidance, a markedly different pattern emerged. When entered first, trust alone explained 21.9% of variance in attachment avoidance (adj. R^2^ = .219, p < .001, β = .300), representing the largest Step 1 contribution of any subscale across all outcomes examined. Credulity and mistrust did not contribute significant additional variance beyond trust in Steps 2 and 3 (Δadj. R^2^ = .004, p = .057 and Δadj. R^2^ = -.001, p = .655 respectively). Conversely, when mistrust was entered first it contributed only marginally (adj. R^2^ = .143, p = .028), credulity added nothing significant (Δadj. R^2^ = −.001, p = .448), and trust retained its large and highly significant incremental contribution in Step 3 (Δadj. R^2^ = .080, β = .300, p < .001) regardless of entry order. The final model explained 22.2% of variance in attachment avoidance (adjusted R^2^ = .222). This pattern indicates that the predictive variance in attachment avoidance is almost entirely carried by the Trust subscale, with mistrust and credulity contributing negligible unique variance once trust is accounted for. For details, see Table 5.

**Table 5:** Incremental validity of ETMCQ-R-6subscales).

Entry Order	Step 1 R² (p)	Step 2 ΔR² (p)	Step 3 ΔR² (p)	Final R²
**General Psychological Distress (BSI Global Severity Index)**				
*Mistrust → Credulity → Trust*	.152 (< .001)	.017 (.001)	.003 (.098)	.172
*Credulity → Mistrust → Trust*	.120 (< .001)	.049 (< .001)	.003 (.098)	.172
*Trust → Credulity → Mistrust*	.075 (.027)	.056 (< .001)	.041 (< .001)	.172
**Borderline Personality Features (PAI-BOR Total Score)**				
*Mistrust → Credulity → Trust*	.288 (< .001)	.068 (< .001)	.002 (.101)	.359
*Credulity → Mistrust → Trust*	.264 (< .001)	.092 (< .001)	.002 (.101)	.359
*Trust → Credulity → Mistrust*	.113 (.026)	.164 (< .001)	.082 (< .001)	.359
**Attachment Anxiety (ECR-R Anxiety Subscale)**				
*Mistrust → Credulity → Trust*	.192 (< .001)	.066 (< .001)	.005 (.049)	.262
*Credulity → Mistrust → Trust*	.221 (< .001)	.037 (< .001)	.005 (.049)	.262
*Trust → Credulity → Mistrust*	.107 (.023)	.125 (< .001)	.029 (< .001)	.262
**Attachment Avoidance (ECR-R Avoidance Subscale)**				
*Mistrust → Credulity → Trust*	.143 (.028)	-.001 (.448)	.080 (< .001)	.222
*Credulity → Mistrust → Trust*	.138 (.138)	.004 (.073)	.080 (< .001)	.222
*Trust → Credulity → Mistrust*	.219 (< .001)	.004 (.057)	-.001 (.655)	.222

Note. All R² values are adjusted R². Step 1 reports the adjusted R² for the model including demographic covariates (gender, age, income, education, marital status) and the first predictor entered. Steps 2 and 3 report the change in adjusted R² (Δadj. R²) relative to the preceding step, with corresponding p-values for the F-change statistic. All three entry orders yield identical final adjusted R² values, confirming that total explained variance is robust to predictor sequence.

## 4 Discussion

The aim of this study was to construct and validate a six-item short form of the revised Epistemic Trust, Mistrust and Credulity Questionnaire (ETMCQ-R) to facilitate use in large-scale epidemiological studies with limited assessment time. Using the same UK quota-balanced community sample that supported the validation of the ETMCQ-R, the results of this analysis provide support for the factorial and conceptual validity, reliability, and comparability of the ETMCQ-R-6 with the initial long-form.

The items for the ETMCQ-R-6 were chosen from the initial 18-item pool of the ETMCQ-R based on their psychometric properties as well as their conceptual relevance. The aim of the item selection process was to select a balance and psychometrically stable set of six items, while acknowledging the potential loss of information that goes along with the construction of a questionnaire short form. The CFA of the ETMCQ-R-6 revealed acceptable model fit, thus confirming the hypothesized three-factor structure consisting of epistemic trust, epistemic mistrust, and epistemic credulity. The fit indices of the ETMCQ-R-6 even outperformed the factorial validity of the initial ETMCQ long form (CFI/TLI: 0.97–0.98 vs. 0.90–0.94; RMSEA: 0.06–0.08 vs. 0.03–0.05) [5] and was comparable with the fit indices for the revised ETMCQ long form (ETMCQ-R) [23]. However, the items were not merely chosen based on their psychometric properties, but also to represent the breadth of each latent construct. For epistemic trust, the two retained items reflect benevolence appraisal (i.e., perceiving others’ input as offered with supportive intent) as well as the relevance of social information provided by others, which in combination capture the core concept of epistemic trust as openness to accepting personally meaningful guidance. The mistrust items represent sensitivity to potential malevolence as well as protective resistance to influence, reflecting the defensive social stance characteristic of epistemic hypervigilance. For credulity, the two items capture complementary aspects of suggestibility as well as over-openness, which aligns with the conceptualizations of credulity as insufficient filtering of social information. Since each factor is assessed by only two items, the potential impact of method effects warrants acknowledgement: the item pair in all subscales share the same response polarity, which may introduce shared method variance such as acquiescence or valence effects. Because each factor is represented by only two indicators, the capacity to detect method effects (e.g., through residual covariances) is limited. Future studies may therefore consider including additional or parallel-worded items with opposite valence to better assess potential method variance.

It should be noted that the upper bound of the RMSEA confidence interval slightly exceeded  .10, indicating that poor model fit cannot be entirely ruled out. This is likely attributable to the relatively small derivation sample, as confidence intervals around RMSEA estimates are strongly influenced by sample size. This interpretation is supported by the independent German replication (N = 2,522), in which the robust RMSEA was  .060 [90% CI:  .047,  .074], with an upper bound well within the acceptable range, suggesting that the three-factor structure achieves adequate fit in larger and more representative samples.

A finding that warrants explicit discussion is the Heywood case observed for the Trust subscale in our sample, which constitutes an inadmissible parameter estimate. Such improper solutions can arise in CFA under conditions of sampling fluctuation or empirical under-identification, and two-indicator factors are particularly vulnerable because they provide limited information for latent construct estimation [47,48]. Importantly, this finding did not replicate in the independent German representative sample (N = 2,522), where the identical six-item model was estimated freely without parameter constraints, all residual variances remained positive, and all standardized loadings were admissible (λ = .57–.89). This cross-sample pattern indicates that the Heywood case in the UK sample was driven by estimation instability in a small derivation sample rather than by structural misspecification of the six-item model. In multi-factor CFA models, two-indicator factors can be identified when embedded in a broader model with correlated latent factors [49,50], which is the case here. Nevertheless, two-indicator subscales remain less robust than three-indicator solutions, and we therefore recommend that the ETMCQ-R-6 be used in samples of adequate size, that subscale scores be interpreted with appropriate caution at the individual level, and that the full 18-item ETMCQ-R be preferred in contexts requiring maximum psychometric precision.

An interesting finding concerns the historical origin of the six items comprising the ETMCQ-R-6. The current validated full-length instrument, the ETMCQ-R [23], represents a revised version of an earlier instrument, the original ETMCQ [5]. When examining which items were retained in the ETMCQ-R-6, it emerged that all six selected items were already present in this earlier original version of the questionnaire, predating the subsequent revision. This historical continuity has a practical implication: researchers who collected data using the ETMCQ or ETMCQ-R may be able to score the ETMCQ-R-6 retrospectively from their existing datasets without requiring new data collection, provided the relevant six items were included. This extends the potential reach of the short form to previously collected datasets and may facilitate longitudinal or cross-study comparisons. The high correlations and excellent congruence between the ETMCQ-R-6 and the full 18-item ETMCQ-R further confirm that the abbreviated version closely preserves the essential psychometric and conceptual properties of the full instrument. These results align with established recommendations suggesting that short forms can maintain validity when item selection is theory-driven and empirically guided [51].

Internal consistency was acceptable for the trust and credulity subscales, while reliability indices fell below the conventional thresholds for the mistrust subscale. This observation carries some practical implications: at the individual level reduced reliability increases measurement errors and thus reduces the precision of individual scores, thus the mistrust subscale of the ETMCQ-R-6 should not be used as a sole basis for important individual-level clinical decisions. Researchers conducting within-person analyses or using the mistrust subscale as a primary individual-level outcome measure should consider using the full 18-item ETMCQ-R, which provides substantially higher subscale reliability. At the group level, measurement error may be averaged out across participants, thus the mistrust subscale retains meaningful utility for research applications examining associations between epistemic mistrust and other constructs. This is underscored by the robust and theoretically coherent correlations with attachment insecurity, borderline features, and psychological distress observed in the present study, as well as its significant incremental predictive validity across both psychopathology outcomes. The relatively low internal consistencies in the ETMCQ-R-6 subscales likely reflects the intentional content heterogeneity of the two retained items, which capture conceptually complementary poles on the construct rather than redundant facets of the measured construct. This represents a deliberate trade-off between content breadth and internal consistency that is inherent to ultra-brief measurement and difficult to avoid while preserving the conceptual range of a multifaceted construct.

To further evaluate the applicability of the ETMCQ-R-6, several aspects of construct validity were tested. For one, we tested the correlation of each ETMCQ-R-6 scale with a broad range of external factors and compared the correlations with the ETMCQ-R long version. To summarize, across all three subscales, correlations with external constructs closely mirrored those of the full ETMCQ-R. The trust subscale was most strongly associated with lower attachment avoidance, more perceived social support and better mentalizing capabilities. Correlations with those key constructs theoretically related to trust remained virtually unchanged compared to the ETMCQ-R trust scale, with average reductions in correlation strength below 10%. The mistrust subscale also showed strong associations with attachment anxiety and avoidance, borderline personality features, and psychological distress, thus retaining robust construct validity. Although minor attenuations (*Δr* ≈ .05–.12) were observed, the direction and relative magnitude of correlations remained highly consistent with the long version of the ETMCQ-R. Similarly, the credulity subscale maintained its expected pattern of positive associations with borderline features and psychological distress, and negative associations with resilience and perceived social support. Slightly larger reductions in correlation strength (up to 25–30%) were observed for some interpersonal measures, yet these changes remained within acceptable limits. The results indicate that even with two items per subscale, the ETMCQ-R-6 preserves the conceptual breadth of the original scale and continues to capture adaptive openness to social communication on the one hand as well as epistemic ruptures, expressed by a tendency toward excessive or uncritical acceptance of information from others or defensive and hypervigilant social attitudes linked to early relational trauma and psychopathology [1,52,53].

To evaluate the incremental validity of each of the ETMCQ-R-6 subscales, hierarchical regression analyses were calculated to determine the additional value in explained variance of each subscale for general psychological distress, borderline symptomatology and attachment styles after controlling for sociodemographic variables. While both epistemic mistrust and credulity were substantially associated with psychopathology features, epistemic trust did not add substantial information to the model. However, for attachment styles a different pattern emerged: the trust subscale alone explained 21.9% of variance in attachment avoidance, which was the largest primary contribution of any subscale across all outcomes, while mistrust and credulity contributed negligible additional variance once trust was accounted for. This pattern of outcome-specific dominance is in line with the theoretical epistemic trust framework: trust operates primarily through interpersonal mechanisms linked to attachment and social openness rather than through direct psychopathology pathways, and its incremental validity is most apparent for outcomes in that domain [4].

These findings align with the broader theoretical framework that conceptualizes epistemic trust as a central mechanism in the development of psychopathology. While earlier models of attachment and mentalizing primarily emphasized the caregiver–child dyad as the key aspect of socio-cognitive development [54], more recent perspectives have expanded this view to incorporate the influence of the broader social environment and the quality of social communication within it [53]. Within this framework, the child’s epistemic stance develops not only in response to primary attachment relationships but also through exposure to mentalizing – or non-mentalizing – contexts in families, schools, and communities. A non-mentalizing social environment can restrict openness to social learning, promoting either entrenched epistemic mistrust or excessive credulity.

We recommend to score each subscale as the mean of its two items, with higher scores indicating higher levels of mistrust, credulity, or trust, respectively. We further recommend to only calculate subscale scores when both items are answered. To support use in research settings, age and gender specific descriptive statistics for each subscale are provided in the Supplementary Materials. We note that the current validation was conducted in a community sample and does not support individual-level clinical decision-making. Before the ETMCQ-R-6 can be recommended for clinical screening or treatment monitoring at the individual level, dedicated clinical validation including test-retest reliability, sensitivity and specificity analyses, and establishment of clinical cut-off scores are required. Since all six items originate from the original ETMCQ, the validation of the ETMCQ-R-6 based on existing datasets is possible without the need to collect new data. Researchers who previously administered the full-scale ETMCQ can directly reconstruct the short-form subscales and evaluate their performance in independent samples, clinical cohorts, or longitudinal studies. Such secondary analyses would offer a valuable test of generalizability and provide additional evidence for the stability and applicability of the shortened measure across contexts. To facilitate replication and retrospective use, a mapping table linking each ETMCQ-R-6 item to the original ETMCQ item number is provided in the Supplements.

Several limitations should be acknowledged. For one, for pragmatic reasons, the validation was conducted using the same dataset as the original ETMCQ-R, and thus independent replication in new international samples is warranted to confirm the generalizability of the findings. This limitation is addressed in part by the independent replication in a nationally representative German general population sample (N = 2,522) reported above, which confirmed the three-factor structure with excellent fit and no inadmissible parameter estimates. The slightly lower internal consistency of the mistrust subscale also suggests that further refinement of item content may be beneficial. In the gender invariance analyses, the original multigroup model produced a Heywood case for the trust factor, which is likely related to the use of only two indicators, and baseline fit in the male subsample fell below conventional thresholds, driven by Trust subscale instability. However, a sensitivity analysis using an equal-loading specification for the trust items yielded admissible estimates and the same substantive pattern of invariance results, and the findings were further replicated in the German sample. This suggests that the gender invariance findings are reasonably robust for the Mistrust and Credulity subscales, although they should still be interpreted with appropriate caution.

Due to the cross-sectional character of the study, no information on test-retest reliability and sensitivity to change could be obtained. Since temporal stability is a prerequisite for instruments intended to monitor change over time, the current design does not allow conclusions about whether the ETMCQ-R-6 produces stable scores in the absence of intervention or whether it is sensitive to genuine change when an intervention is delivered. It also does not permit estimation of the magnitude of score change that would represent a clinically meaningful difference beyond measurement error. Establishing the temporal stability and responsiveness of the ETMCQ-R-6 therefore represents a priority for future research. Until such data are available, the instrument should be used for cross-sectional research and population-level assessment rather than for individual-level monitoring of change over time.

No dedicated attention check items or instructed-response questions were included in the survey design. Although Prolific has been shown to yield substantially lower rates of careless responding compared with other online recruitment platforms [55], the absence of formal careless responding screening represents a limitation of the current study. The observed psychometric properties of the data – including adequate item-level variability, expected inter-factor correlations, and theoretically coherent external validity patterns – are inconsistent with substantial contamination from random responding, but future studies using the ETMCQ-R-6 in online contexts should incorporate instructed-response items to allow formal careless responding screening [56]. The item selection procedure, while transparent and theory-guided, relied on stepwise CFA-based deletion rather than modern metaheuristic optimization approaches such as Ant Colony Optimization or Genetic Algorithms [36], which represents a further methodological limitation. Future research should therefore focus to examine cross-cultural invariance, test-retest reliability, and predictive validity in both clinical and non-clinical populations.

In summary, the results indicate that the ETMCQ-R-6 is a practically useful brief screening measure that retains key structural and construct validity characteristics of the ETMCQ-R and provides an efficient tool for assessing epistemic trust, mistrust, and credulity across diverse research settings.

## Supporting information

S1 Table External validity summary table. Pearson correlations and Tucker’s congruence coefficients between the ETMCQ-R and ETMCQ-R-6 subscales and all external validity measures across trust, mistrust, and credulity subscales.
(DOCX)

S2 Table Descriptive statistics and percentile-based cutoffs for ETMCQ-R-6 subscales by age group and gender in the UK community sample (N = 506). Reports means, standard deviations, and 25th, 50th, and 75th percentile scores stratified by gender and age group.
(DOCX)

S3 Table Factor loadings, communalities, and corrected item-total correlations for all 18 items of the ETMCQ-R and the six items of the ETMCQ-R-6 in the UK derivation sample. (DOCX)

S4 Table Comprehensive measurement invariance table. Reports scaled χ², df, p-value, robust CFI, robust TLI, robust RMSEA with 90% CI, SRMR, Δχ², ΔCFI, and ΔRMSEA for all invariance model steps across gender and age groups, including sensitivity analyses and baseline within-group CFAs.
(DOCX)
