# Supplementary material for: Can we trust this short scale? Validation of a Brief Self-Report Measure of Epistemic Trust, Mistrust and Credulity (ETMCQ-R-6) in a sample of the U.K. general population
Source: PLOS Ment Health. 2026 Aug 31;3(8):e0000591. doi: 10.1371/journal.pmen.0000591 (PMC13529005; doi:10.1371/journal.pmen.0000591)
Supplement: Supplementary file 1 [file pmen.0000591.s001.docx]

S1 Table: External validity summary table

|  | **Trust subscale** | | | | **Mistrust subscale** | | | | **Credulity subscale** | | | |
| --- | --- | --- | --- | --- | --- | --- | --- | --- | --- | --- | --- | --- |
|  | **r**  **(full)** | **r**  **(short)** | **Δr** | **q** | **r  (full)** | **r  (short)** | **Δr** | **q** | **r  (full)** | **r (short)** | **Δr** | **q** |
| ECR-R Anxiety | -.06 | -.09 | -.03 | .04 | .45 | .33 | -.12 | .13 | .48 | .39 | -.09 | .11 |
| ECR-R Avoidance | .37 | .35 | -.02 | .02 | -.13 | -.09 | .04 | .04 | -.14 | -.07 | .07 | .07 |
| MZQ Total | .15 | .18 | .03 | .03 | -.60 | -.48 | .12 | .17 | -.58 | -.44 | .14 | .20 |
| BSI Depression | -.07 | -.10 | -.03 | .04 | .39 | .32 | -.07 | .07 | .37 | .26 | -.11 | .12 |
| BSI Anxiety | .02 | .02 | 0 | .01 | .39 | .31 | -.08 | .09 | .38 | .30 | -.08 | .10 |
| BRS | .06 | .08 | .02 | .02 | .23 | .21 | -.02 | .02 | .25 | .22 | -.03 | .03 |
| MSPSS Total | .41 | .41 | 0 | .00 | -.32 | -.23 | .09 | .10 | -.23 | -.12 | .11 | .11 |
| MSPSS Significant Other | .24 | .26 | .02 | .02 | -.18 | -.13 | .05 | .05 | -.17 | -.10 | .07 | .07 |
| MSPSS Family | .32 | .33 | .01 | .01 | -.27 | -.18 | .09 | .10 | -.21 | -.11 | .10 | .10 |
| MSPSS Friends | .45 | .41 | -.04 | .04 | -.33 | -.25 | .08 | .09 | -.19 | -.08 | .11 | .11 |
| PAI-BOR Total | -.04 | -.03 | .01 | .00 | .54 | .46 | -.08 | .11 | .55 | .48 | -.07 | .09 |
| EVS Indifference | -.10 | .01 | .11 | .12 | .28 | .20 | -.08 | .08 | .31 | .26 | -.05 | .06 |
| EVS Rigidity | -.09 | -.01 | .08 | .08 | .23 | .15 | -.08 | .09 | .19 | .19 | 0 | .00 |
