# Supplementary material for: Can we trust this short scale? Validation of a Brief Self-Report Measure of Epistemic Trust, Mistrust and Credulity (ETMCQ-R-6) in a sample of the U.K. general population
Source: PLOS Ment Health. 2026 Aug 31;3(8):e0000591. doi: 10.1371/journal.pmen.0000591 (PMC13529005; doi:10.1371/journal.pmen.0000591)
Supplement: Supplementary file 2 [file pmen.0000591.s002.docx]

| **S2 Table: Descriptive statistics for ETMCQ-R-6 subscales by age group and gender (UK sample, N = 506)** | | | | | | | |
| --- | --- | --- | --- | --- | --- | --- | --- |
|  |  | **Trust** | | **Mistrust** | | **Credulity** | |
|  | **N** | **M** | **SD** | **M** | **SD** | **M** | **SD** |
| **< 30 years** |  |  |  |  |  |  |  |
| Female | 54 | 5.34 | 1.05 | 4.11 | 1.15 | 2.95 | 1.29 |
| Male | 54 | 4.35 | 1.21 | 4.31 | 1.1 | 2.68 | 1.19 |
| Total | 108 | **4.85** | 1.23 | **4.21** | 1.13 | **2.81** | 1.24 |
| **30–40 years** |  |  |  |  |  |  |  |
| Female | 54 | 4.79 | 1.35 | 4.27 | 1.09 | 3.01 | 1.52 |
| Male | 45 | 4.39 | 1.36 | 4 | 1.26 | 2.53 | 1.24 |
| Total | 99 | **4.61** | 1.36 | **4.15** | 1.17 | **2.79** | 1.41 |
| **40–50 years** |  |  |  |  |  |  |  |
| Female | 44 | 4.47 | 1.34 | 4.18 | 1.33 | 2.97 | 1.36 |
| Male | 40 | 3.93 | 1.6 | 4.18 | 1.48 | 2.39 | 1.24 |
| Total | 84 | **4.21** | 1.49 | **4.18** | 1.39 | **2.69** | 1.33 |
| **50–60 years** |  |  |  |  |  |  |  |
| Female | 56 | 4.88 | 1.25 | 4.09 | 1.37 | 2.44 | 1.31 |
| Male | 47 | 4.22 | 1.37 | 3.71 | 1.31 | 2.28 | 1.21 |
| Total | 103 | **4.58** | 1.34 | **3.92** | 1.35 | **2.36** | 1.26 |
| **> 60 years** |  |  |  |  |  |  |  |
| Female | 58 | 4.35 | 1.39 | 3.71 | 1.12 | 2.23 | 0.98 |
| Male | 54 | 4.19 | 1.49 | 3.43 | 0.97 | 2.25 | 1.05 |
| Total | 112 | **4.28** | 1.44 | **3.57** | 1.06 | **2.24** | 1.01 |
| **Overall sample** |  |  |  |  |  |  |  |
| Female | 266 | 4.77 | 1.32 | 4.06 | 1.22 | 2.7 | 1.33 |
| Male | 240 | 4.23 | 1.4 | 3.91 | 1.25 | 2.43 | 1.18 |
| **Total** | 506 | **4.51** | 1.38 | **3.99** | 1.24 | **2.57** | 1.27 |
| *M = mean; SD = standard deviation; higher scores indicate greater endorsement of each epistemic stance (trust, mistrust, credulity).* | | | | | | | |

| **Percentile-Based Cutoffs for the ETMCQ-R-6 Subscales** | | | | | | | | | | | |
| --- | --- | --- | --- | --- | --- | --- | --- | --- | --- | --- | --- |
| **Group** | **N** | **Epistemic Trust** | | | **Epistemic Mistrust** | | | **Epistemic Credulity** | | | |
|  |  | ***P25*** | ***P50*** | ***P75*** | ***P25*** | ***P50*** | ***P75*** | ***P25*** | ***P50*** | ***P75*** |  |
| Full sample | 515 | 3.50 | 4.50 | 5.50 | 3.00 | 4.00 | 5.00 | 1.50 | 2.00 | 3.50 |  |
| **By Gender** |  |  |  |  |  |  |  |  |  |  |  |
| Female (n = 266) | 266 | 4.00 | 5.00 | 5.88 | 3.50 | 4.00 | 5.00 | 2.00 | 2.50 | 3.50 |  |
| Male (n = 240) | 240 | 3.50 | 4.50 | 5.50 | 3.00 | 4.00 | 5.00 | 1.50 | 2.00 | 3.00 |  |
| **By Age** |  |  |  |  |  |  |  |  |  |  |  |
| <30 years (n = 113) | 113 | 4.00 | 5.00 | 5.50 | 3.50 | 4.00 | 5.00 | 2.00 | 2.50 | 4.00 |  |
| 30–39 years (n = 100) | 100 | 3.50 | 4.50 | 6.00 | 3.50 | 4.00 | 5.00 | 2.00 | 2.50 | 4.00 |  |
| 40–49 years (n = 85) | 85 | 3.00 | 4.50 | 5.50 | 3.50 | 4.00 | 5.00 | 1.50 | 2.50 | 3.50 |  |
| 50–59 years (n = 105) | 105 | 4.00 | 5.00 | 5.50 | 3.00 | 4.00 | 4.50 | 1.50 | 2.00 | 3.00 |  |
| ≥60 years (n = 112) | 112 | 3.00 | 4.50 | 5.50 | 3.00 | 3.50 | 4.00 | 1.50 | 2.00 | 3.00 |  |
