# Supplementary material for: Can we trust this short scale? Validation of a Brief Self-Report Measure of Epistemic Trust, Mistrust and Credulity (ETMCQ-R-6) in a sample of the U.K. general population
Source: PLOS Ment Health. 2026 Aug 31;3(8):e0000591. doi: 10.1371/journal.pmen.0000591 (PMC13529005; doi:10.1371/journal.pmen.0000591)
Supplement: Supplementary file 3 [file pmen.0000591.s003.docx]

| S3 Table: Factor loadings, communalities and item-total correlations for the ETMCQ-R and ETMCQ-R-6 items | | | | | | | | | | |
| --- | --- | --- | --- | --- | --- | --- | --- | --- | --- | --- |
|  |  |  |  |  | Factor loading | | Communalities | | Corrected item-total correlation | |
| Nr ^1^ | Item | Subscale | Mean | SD | 18-item version | 6-item version | 18-item version | 6-item version | 18-item version | 6-item version |
| 1 (1) | I usually ask people for advice when I have a personal problem. | Trust | 4.17 | 1.61 | 0.70 | 1.00 | 0.49 | 1.00 | 0.56 | 0.60 |
| 2 | I find information easier to trust and absorb when it comes from someone who knows me well | Trust | 5.28 | 1.21 | 0.68 | -- | 0.47 | -- | 0.59 | -- |
| 3 | I often feel that people do not understand what I want and need | Mistrust | 4.48 | 1.54 | 0.62 | -- | 0.38 | -- | 0.53 | -- |
| 4 | I am often considered naïve because I believe almost anything that people tell me | Credulity | 2.73 | 1.62 | 0.67 | -- | 0.45 | -- | 0.63 | -- |
| 5 | Sometimes, having a conversation with people who have known me for a long time helps me develop new perspectives about myself | Trust | 4.85 | 1.30 | 0.54 | -- | 0.29 | -- | 0.49 | -- |
| 6 | I find it very useful to learn from what people tell me about their experiences | Trust | 5.30 | 1.05 | 0.55 | -- | 0.31 | -- | 0.53 | -- |
| 7 (2) | If you put too much faith in what people tell you, you are likely to get hurt | Mistrust | 4.30 | 1.39 | 0.64 | 0.75 | 0.41 | 0.56 | 0.58 | 0.51 |
| 8 (3) | When someone tells me something, my immediate reaction is to wonder why they are telling me this. | Mistrust | 3.66 | 1.46 | 0.65 | 0.69 | 0.42 | 0.47 | 0.58 | 0.51 |
| 9 (4) | I have too often taken advice from the wrong people | Credulity | 2.66 | 1.31 | 0.71 | 0.84 | 0.50 | 0.70 | 0.64 | 0.63 |
| 10 (5) | People have told me that I am too easily influenced by others | Credulity | 2.49 | 1.50 | 0.76 | 0.75 | 0.58 | 0.56 | 0.72 | 0.63 |
| 11 (6) | If I don’t know what to do, my first instinct is to ask someone whose opinion I value | Trust | 4.87 | 1.48 | 0.72 | 0.56 | 0.52 | 0.31 | 0.59 | 0.60 |
| 12 | In the past, I have misjudged who to believe and been taken advantage of | Credulity | 3.18 | 1.50 | 0.79 | -- | 0.63 | -- | 0.72 | -- |
| 13 | I frequently feel that I don’t know what to think about what someone is telling me, even when I understand what they said | Credulity | 3.45 | 1.46 | 0.71 | -- | 0.50 | -- | 0.62 | -- |
| 14 | I am genuinely curious about other people’s views | Trust | 5.31 | 1.22 | 0.61 | -- | 0.15 | -- | 0.35 | -- |
| 15 | When someone is trying to explain something that they think is important, I often feel that what they are saying is not relevant to me | Mistrust | 3.71 | 1.32 | 0.59 | -- | 0.35 | -- | 0.53 | -- |
| 16 | When someone is telling me something, I tend to feel like they’re forcing their ideas onto me | Mistrust | 3.20 | 1.32 | 0.63 | -- | 0.39 | -- | 0.53 | -- |
| 17 | I often feel blank in situations where other people seem to have a clear view about what they are being told | Credulity | 3.46 | 1.46 | 0.61 | -- | 0.37 | -- | 0.56 | -- |
| 18 | People don’t usually care about being truthful in what they say | Mistrust | 3.57 | 1.39 | 0.68 | -- | 0.47 | -- | 0.59 | -- |
| ^1^ Numbers in brackets are item numbers for the ETMCQ-R-6 | | | | | | | | | | |
