# Supplementary material for: Can we trust this short scale? Validation of a Brief Self-Report Measure of Epistemic Trust, Mistrust and Credulity (ETMCQ-R-6) in a sample of the U.K. general population
Source: PLOS Ment Health. 2026 Aug 31;3(8):e0000591. doi: 10.1371/journal.pmen.0000591 (PMC13529005; doi:10.1371/journal.pmen.0000591)
Supplement: Supplementary file 4 [file pmen.0000591.s004.docx]

| S4 Table: Measurement Invariance Analyses | | | | | | | | | | | | |
| --- | --- | --- | --- | --- | --- | --- | --- | --- | --- | --- | --- | --- |
| **Model** | **χ² (scaled)** | **df** | **p** | **CFI robust** | **TLI robust** | **RMSEA robust [90% CI]** | **SRMR** | **Δχ²** | **Δdf** | **p(Δ)** | **ΔCFI** | **ΔRMSEA** |
| **Gender Invariance** | | | | | | | | | | | | |
| Baseline: Females | 9.83 | 6 | .132 | .991 | .978 | .048 [.000, .101] | .031 | — | — | — | — | — |
| Baseline: Males | 67.74 | 6 | .000 | .944 | .859 | .109 [.087, .133] | .045 | — | — | — | — | — |
| Configural | 45.28 | 12 | .000 | .971 | .928 | .083 [.058, .109] | .033 | — | — | — | — | — |
| Metric | 28.07 | 15 | .021 | .981 | .961 | .061 [.023, .095] | .035 | 0.57 | 3 | .904 | .010 | .022 |
| Scalar | 33.04 | 18 | .017 | .978 | .963 | .059 [.025, .091] | .037 | 4.95 | 3 | .176 | .003 | .002 |
| Structural | 35.92 | 24 | .056 | .982 | .978 | .046 [.000, .076] | .046 | 3.25 | 6 | .777 | .004 | .013 |
| Strict | 53.67 | 30 | .005 | .964 | .964 | .059 [.032, .084] | .058 | 17.06 | 6 | .009 | .018 | .013 |
| **Gender Invariance — Sensitivity Analysis: Equal Trust Loadings** | | | | | | | | | | | | |
| Configural | 35.13 | 15 | .002 | .972 | .943 | .073 [.042, .105] | .036 | — | — | — | — | — |
| Metric | 34.25 | 16 | .005 | .973 | .948 | .070 [.037, .102] | .038 | 0.72 | 1 | .398 | .001 | .003 |
| Scalar | 41.76 | 19 | .002 | .966 | .947 | .071 [.042, .100] | .040 | 7.65 | 3 | .054 | .007 | .001 |
| Structural | 43.99 | 25 | .011 | .972 | .966 | .057 [.027, .084] | .049 | 2.81 | 6 | .832 | .006 | .014 |
| Strict | 60.75 | 31 | .001 | .954 | .956 | .065 [.040, .089] | .060 | 16.26 | 6 | .012 | .018 | .008 |
| **Age Invariance** | | | | | | | | | | | | |
| Baseline: <30 years | 12.63 | 6 | .049 | .926 | .814 | .100 [.005, .177] | .056 | — | — | — | — | — |
| Baseline: 30–39 years | 8.29 | 6 | .217 | .982 | .954 | .068 [.000, .168] | .060 | — | — | — | — | — |
| Baseline: 40–49 years | 1.68 | 6 | .946 | 1.000 | 1.095 | .000 [.000, .020] | .021 | — | — | — | — | — |
| Baseline: 50–59 years | 5.01 | 6 | .542 | 1.000 | 1.014 | .000 [.000, .124] | .044 | — | — | — | — | — |
| Baseline: ≥60 years | NA | 6 | NA | NA | NA | NA | .041 | — | — | — | — | — |
| Configural | 42.40 | 30 | .066 | .985 | .962 | .060 [.000, .098] | .040 | — | — | — | — | — |
| Metric | 53.83 | 42 | .104 | .984 | .972 | .051 [.000, .088] | .049 | 12.41 | 12 | .413 | .001 | .009 |
| Scalar | 67.80 | 54 | .098 | .981 | .973 | .050 [.000, .084] | .054 | 14.15 | 12 | .291 | .003 | .001 |
| Structural | 101.40 | 78 | .039 | .966 | .967 | .056 [.014, .084] | .115 | 33.25 | 24 | .099 | .015 | .006 |
| Strict | 147.78 | 102 | .002 | .932 | .950 | .069 [.042, .092] | .125 | 45.69 | 24 | .005 | .034 | .013 |
| Note. Δ statistics reflect comparison to the preceding model. CFI = Comparative Fit Index; RMSEA = Root Mean Square Error of Approximation; SRMR = Standardized Root Mean Square Residual; CI = Confidence Interval. Invariance criteria: ΔCFI ≤ .010; ΔRMSEA < .015. Baseline CFAs estimated separately within each group prior to multigroup modelling. For the ≥60 age group, fit indices were not available (NA) due to a non-positive definite covariance matrix, consistent with Trust subscale instability in that subgroup | | | | | | | | | | | | |
