# Supplementary material for: Can we trust this short scale? Validation of a Brief Self-Report Measure of Epistemic Trust, Mistrust and Credulity (ETMCQ-R-6) in a sample of the U.K. general population
Source: PLOS Ment Health. 2026 Aug 31;3(8):e0000591. doi: 10.1371/journal.pmen.0000591 (PMC13529005; doi:10.1371/journal.pmen.0000591)
Supplement: Supplementary file 5 [file pmen.0000591.s006.docx]

| Journal Requirements: |  |
| --- | --- |
| 1. Please amend your detailed Financial Disclosure statement. This is published with the article. It must therefore be completed in full sentences and contain the exact wording you wish to be published. i. Please clarify all sources of financial support for your study. List the grants, grant numbers, and organizations that funded your study, including funding received from your institution. Please note that suppliers of material support, including research materials, should be recognized in the Acknowledgements section rather than in the Financial Disclosure.  ii. State the initials, alongside each funding source, of each author to receive each grant. For example: "This work was supported by the National Institutes of Health (####### to AM; ###### to CJ) and the National Science Foundation (###### to AM)."  iii. State what role the funders took in the study. If the funders had no role in your study, please state: “The funders had no role in study design, data collection and analysis, decision to publish, or preparation of the manuscript.”  iv. If any authors received a salary from any of your funders, please state which authors and which funders. | Professor Fonagy is contractually required by his funder to include a statement of acknowledgement using exact wording. This is because he is financially supported by this funder across his work rather than in relation to this particular study. Therefore, in this instance we request the following text be included in the acknowledgments.  “This report is independent research supported by the National Institute for Health and Care Research ARC North Thames. The views expressed in this publication are those of the author(s) and not necessarily those of the National Institute for Health and Care Research or the Department of Health and Social Care.“ |
| 2. In the online submission form, you indicated that “The data that support the findings of this study are available from the corresponding author, D.R. upon reasonable request”.  All PLOS journals now require all data underlying the findings described in their manuscript to be freely available to other researchers, either  1. In a public repository,  2. Within the manuscript itself, or  3. Uploaded as supplementary information.  This policy applies to all data except where public deposition would breach compliance with the protocol approved by your research ethics board. If your data cannot be made publicly available for ethical or legal reasons (e.g., public availability would compromise patient privacy), please explain your reasons by return email and your exemption request will be escalated to the editor for approval. Your exemption request will be handled independently and will not hold up the peer review process, but will need to be resolved should your manuscript be accepted for publication. One of the Editorial team will then be in touch if there are any issues. | Thank you for bringing up this point. We have revised the section, which now reads  The research data supporting this publication are stored at our institutional digital data repository for published research accessible via <https://creed.lbg.ac.at> (accessed on June 23^rd^ 2026). The data sets analyzed in this manuscript are not publicly available due to ethical and legal restrictions (data contain potentially identifying and sensitive patient information). However, pseudonymized data sets have been created for the purpose of re-use and are also accessible via <https://creed.lbg.ac.at>. Requests for access to anonymized data sets should be directed to the corresponding author (D.R.). |
| 3. Please upload separate figure files in .tif or .eps format. Also, remove the figures from your manuscript file but keep the legends. | Corrected as suggested. |
| 4. We have noticed that you have uploaded Supporting Information files, but you have not included a list of legends. Please add a full list of legends for your Supporting Information files after the references list. | Corrected as suggested. |

| Reviewer #1: This manuscript describes the development of the ETMCQ-R-6, a six-item short form of the Revised Epistemic Trust, Mistrust and Credulity Questionnaire, derived from the full 18-item ETMCQ-R using a UK quota-balanced community sample (N = 515). The authors selected two items per subscale based on CFA, internal consistency, and content coverage. They report acceptable model fit, adequate reliability for trust and credulity but marginal reliability for mistrust (α = 0.68), configural/metric/structural invariance across gender, and mediation patterns consistent with prior work. The paper concludes that the ETMCQ-R-6 is a psychometrically stable brief screener for research and clinical use.  My recommendation is Major Revisions. The manuscript suffers from two foundational flaws that cannot be resolved within the current framework merely by disclosing them as limitations.  First, the short form is developed and tested on the identical sample used for the parent ETMCQ-R validation, which maximally capitalizes on sample-specific variance and artificially inflates apparent psychometric correspondence.  Second, the decision to construct each factor from only two items directly transgresses the widely accepted three-indicator rule for latent variable models (Hair et al., 2019), producing an inadmissible Heywood case (β = 1.09 for a trust item) and a second borderline Heywood case in the holdout sample. Because of these structural limitations, the paper does not provide credible evidence that the ETMCQ-R-6 functions as a valid short form. I believe a revision is only acceptable if the authors (a) reconceptualize the short form as a nine-item instrument (i.e., at least three items per subscale) to satisfy basic identification requirements, and (b) validate this new short form with at least some new, independent data that were not used in the development of the parent ETMCQ-R. The detailed comments below elaborate on these and additional issues that must be addressed. |  |
| --- | --- |
| Major Comments  1. The manuscript states that the data were collected between March 16 and March 23, 2023, from 525 adults via Prolific. This is the exact same dataset that supported the validation of the parent 18-item ETMCQ-R instrument. Deriving a short form and conducting all psychometric analyses on the very same dataset used to validate the parent instrument is a critical methodological error. Smith et al. (2000), in their authoritative paper on short-form development, explicitly warn against this practice, as it capitalizes on sample-specific covariance and leads to overoptimistic estimates of factor loadings, model fit, and short-to-long-form correspondence. The internal 70/30 holdout split does not remedy this, as the data source remains identical. Disclosing this as a limitation is insufficient for publication in a top-tier journal. The authors must commit to validating the short form using a genuinely independent sample. | We fully agree with the reviewer that developing and validating a short form within the same dataset used for the parent instrument is a methodological limitation. We acknowledge that the 70/30 holdout split, while useful as an internal check, does not constitute independent validation.  To address this directly, we present validation of the ETMCQ-R-6 in a genuinely independent sample: a nationally representative German general population survey (N = 2,522; Riedl et al., under review). This dataset was collected independently, uses a random route sampling design for representativeness, and was not involved in any stage of the ETMCQ-R or ETMCQ-R-6 development.  In this independent German sample, confirmatory factor analysis using the identical 6-item structure and MLR estimation (as recommended by the reviewer in Comment 3) demonstrated excellent model fit: χ²(6) = 58.74, p < .001; CFI = .984; TLI = .961; RMSEA = .060 [90% CI: .047, .074]; SRMR = .069. All standardized factor loadings were admissible, ranging from λ = .57 to λ = .89, and no Heywood cases or inadmissible parameter estimates occurred. The three-factor structure replicated cleanly across this independent large-scale representative sample, providing strong evidence that the psychometric properties observed in the UK derivation sample are not sample-specific artifacts.  We have added a subsection reporting this independent replication to the Results section and have cited this evidence prominently in the Discussion. |
| 2. The standardized loading of 1.09 for Trust Item #1 is a Heywood case and an inadmissible solution. This is not a minor problem that can be explained by noting the item is "highly saturated"; it is a structural inevitability of specifying a factor with only two indicators. The three-indicator rule (Hair et al., 2019) explicitly warns that two-indicator factors are locally under-identified and highly fragile. The authors themselves report that the latent construct reliability (H) for trust reached 1.0; a direct mathematical artifact of the Heywood case that cannot be interpreted as perfect reliability. Furthermore, in the 30% holdout sample, a mistrust loading reached 1.00 (β = 0.51–1.00), proving that another two-item factor became unstable. These findings unequivocally show that the six-item, two-indicators-per-factor model is psychometrically unsound. While constraining the trust loadings eliminates the Heywood case computationally, the underlying structural flaw remains: two-item factors fail to provide adequate construct coverage and produce unreliable parameter estimates. In my view, the authors must abandon the current six-item version and develop a nine-item short form (i.e., at least three items per subscale) to achieve proper identification and genuine content sampling. | We thank the reviewer for this technically precise critique. We agree that the standardized loading of β = 1.09 in the UK derivation sample constitutes a genuine Heywood case and therefore an inadmissible parameter estimate. We have removed the earlier language characterizing this finding as merely reflecting "high saturation." We also agree that two-indicator factors are more vulnerable to empirical identification problems than three-indicator factors and now discuss this limitation explicitly.  However, we respectfully maintain that the Heywood case reflects a sample-specific estimation problem rather than a fundamental structural flaw of the ETMCQ-R-6. In the independent German representative sample (N = 2,522), the identical six-item, two-indicator-per-factor model was estimated freely using MLR without parameter constraints. No Heywood case occurred, all residual variances were positive, and all standardized loadings were admissible (λ = .57–.89). This cross-sample pattern suggests that the inadmissible loading in the smaller UK derivation sample (N = 515) was driven by estimation instability rather than by inevitable misspecification of the six-item structure.  We also agree that three indicators per factor are generally preferable. At the same time, the SEM literature does not treat two-indicator factors as categorically inadmissible. In multi-factor CFA models, two-indicator factors can be identified when embedded in a broader model with correlated latent factors, although they remain less robust and require empirical corroboration (Bollen, 1989; Kline, 2016). This is the case here: Trust, Mistrust, and Credulity are modeled as correlated constructs, and the German replication provides direct evidence that the six-item model can produce stable and admissible estimates in a large independent sample.  We considered the reviewer's suggestion to develop a nine-item version. However, the ETMCQ-R-6 was designed specifically for contexts where brevity is essential, such as population surveys, longitudinal cohorts, and multi-measure clinical or epidemiological batteries. A nine-item version would reduce the original 18-item scale by only 50%, whereas the six-item form provides a substantially greater reduction in participant burden. We therefore retain the six-item version, but now present it more explicitly as a brief-form instrument whose use should be supported by adequate sample size and whose subscale scores should be interpreted with appropriate caution |
| 3. The authors used maximum likelihood (ML) estimation with bootstrapped confidence intervals for factor loadings but retained standard ML for the model χ², CFI, TLI, RMSEA, and SRMR. Given that the items are ordinal (7-point Likert scales) and potential non-normality was acknowledged, this approach is inadequate. Bootstrapping loadings does not correct the model-level test statistic, nor does it provide robust standard errors for all parameters. The appropriate estimators for ordinal indicators are WLSMV (which makes no distributional assumptions and uses polychoric correlations) or MLR. All CFA and invariance analyses must be re-estimated with a robust estimator, and the corresponding adjusted fit indices must be reported. | We agree fully with the reviewer. The original approach of bootstrapping confidence intervals for factor loadings while retaining standard ML for model-level fit statistics was inconsistent and inadequate for ordinal Likert-scale data. We have therefore re-estimated all CFA models and measurement invariance analyses — including the initial 18-item item reduction CFA, the six-item validation CFA, the holdout validation, and all multigroup invariance models — using the robust Maximum Likelihood estimator (MLR) with Yuan-Bentler scaling correction throughout. MLR provides scaled χ² test statistics corrected for non-normality, robust standard errors based on the sandwich estimator, and robust versions of CFI, TLI, and RMSEA appropriate for ordinal Likert-scale responses. We selected MLR over WLSMV because our items use a 7-point response scale, for which ML-based estimators have been shown to perform well (Rhemtulla et al., 2012), and because MLR allows direct comparability with the independent German replication sample, which was also estimated using MLR. All fit indices reported in the revised manuscript reflect MLR estimation throughout: the scaled χ², robust CFI, robust TLI, robust RMSEA with 90% confidence interval, and SRMR. Bootstrapped confidence intervals for loadings have been replaced by MLR sandwich standard errors. All tables, figures, and in-text statistics have been updated accordingly.  Under MLR estimation, the three-factor model demonstrated acceptable fit in the UK derivation sample: scaled χ²(6) = 22.13, p = .001; robust CFI = .978; robust TLI = .945; robust RMSEA = .073 [90% CI: .042, .107]; SRMR = .029. The Heywood case for ETMCQ_01 persisted under MLR (β = 1.085, residual variance = −0.460), as discussed in our response to Comment 2. In the independent German representative sample (N = 2,522), MLR estimation converged cleanly with no inadmissible parameters and excellent fit (CFI = .984, RMSEA = .060), confirming that the instability is sample-specific rather than a consequence of the estimator. |
| 4. The invariance section contains multiple errors and omissions: • The framework is incorrectly described: constraints are cumulative. Metric invariance constrains structure and loadings; scalar constrains structure, loadings, and intercepts; etc. The manuscript implies each step operates independently. • The scalar invariance step is mentioned, but its results (including ΔCFI and Δχ²) are completely absent from the text. • The abstract and methods claim that invariance across age groups was tested, yet no age-invariance results appear anywhere in the results section. • The invariance criteria are incomplete: the authors mention ΔCFI ≤ .010 (Cheung & Rensvold, 2002) but omit the corresponding ΔRMSEA guideline (e.g., ΔRMSEA < .015; Chen, 2007). • Best practice requires testing the CFA model separately within each group (e.g., females and males) to confirm adequate baseline fit before proceeding to multi-group configural invariance. • A comprehensive table is required, listing χ², df, CFI, RMSEA, and the Δχ², ΔCFI, and ΔRMSEA for each model step. | We thank the reviewer for identifying these errors and omissions systematically. All have been fully corrected in the revised manuscript. We address each point in turn.  **Cumulative constraint framework**. The invariance testing sequence has been rewritten to correctly characterize the cumulative constraint logic. The revised text explicitly states that each step adds constraints to all preceding steps: configural (factor structure freely estimated across groups), metric (factor loadings additionally constrained equal), scalar (item intercepts additionally constrained equal), structural (factor variances and covariances additionally constrained equal), and strict (residual variances additionally constrained equal). The previous implication that each step operated independently has been removed.  **Scalar invariance results**. Scalar invariance results are now fully reported for both gender and age analyses, including Δχ², ΔCFI, and ΔRMSEA at each step. For gender, scalar invariance was supported (Δχ²(3) = 4.95, p = .176, ΔCFI = .003, ΔRMSEA = .002). For age, scalar invariance was similarly supported (Δχ²(12) = 14.15, p = .291, ΔCFI = .003, ΔRMSEA = .001).  **Age invariance results**. Age invariance analyses across five age groups (<30, 30–39, 40–49, 50–59, ≥60 years) are now fully reported in the Results section. Configural, metric, and scalar invariance were supported across age groups. Structural invariance was borderline (Δχ²(24) = 33.25, p = .099, ΔCFI = .015, ΔRMSEA = .006) and strict invariance was not supported (Δχ²(24) = 45.69, p = .005, ΔCFI = .034, ΔRMSEA = .013).  ΔRMSEA criterion. The ΔRMSEA < .015 criterion (Chen, 2007) has been added alongside ΔCFI ≤ .010 (Cheung & Rensvold, 2002) throughout the invariance section and in the comprehensive table. Chen (2007) has been added to the reference list.  **Baseline within-group CFAs**. Baseline CFAs were conducted separately within each gender group and each age group prior to multigroup modelling, and results are now reported in the Results section. For gender, baseline fit was acceptable in females (robust CFI = .991, RMSEA = .048 [.000, .101]) but fell below conventional thresholds in males (robust CFI = .944, RMSEA = .109 [.087, .133]). We wish to be fully transparent that this misfit in males is localized to the Trust subscale, where the Heywood case is more pronounced in the male subgroup. A sensitivity analysis using an equality constraint on the two Trust item loadings partially improved male baseline fit (robust CFI = .959, RMSEA = .086), though fit remained below conventional thresholds. We have added an explicit caveat to the Results noting that gender invariance findings should be interpreted with caution in light of inadequate male baseline fit, and that the invariance conclusions are most applicable to the Mistrust and Credulity subscales.  For age, baseline fit was acceptable in the 30–39, 40–49, 50–59, and ≥60 year groups, with the <30 year group showing marginal fit (robust CFI = .926, RMSEA = .100) and the ≥60 year group producing inadmissible fit indices due to a non-positive definite covariance matrix consistent with Trust subscale instability.  **Comprehensive invariance table**. A comprehensive invariance table (Appendix D) has been added to the revised manuscript, listing scaled χ², df, p-value, robust CFI, robust TLI, robust RMSEA with 90% CI, SRMR, and Δχ², ΔCFI, and ΔRMSEA for every model step across both gender and age invariance analyses, including the sensitivity analysis with equal Trust loadings and all baseline within-group CFAs.  **Independent replication of invariance**. As additional cross-sample evidence, we note that the same full MLR-based invariance sequence was conducted in the independent German representative sample (N = 2,522). Configural and metric invariance were replicated across gender and age in the German sample, consistent with the UK findings. This cross-sample replication of the factor loading equivalence strengthens confidence in the structural comparability of the ETMCQ-R-6 across demographic groups. |
| 5. Data were collected online via Prolific, yet the manuscript says nothing about attention checks, instructed-response items, or mechanisms for identifying careless respondents. Given the well-documented threat of inattentive responding in unsupervised web-based samples (Ward & Meade, 2023), this is a serious omission. The lack of screening may have artificially inflated reliability estimates and distorted CFA results. This must be detailed if employed, or heavily acknowledged as a major threat to data quality. | We thank the reviewer for raising this important methodological point. We acknowledge that the original manuscript did not describe any attention check procedures, and we address this transparently below.  No dedicated instructed-response items or formal attention check questions were included in the parent ETMCQ-R validation study (Campbell et al., 2025), which used the identical dataset and similarly did not report attention check procedures. We acknowledge that the absence of formal careless responding screening is a limitation that applies to both studies.  However, several features of the study design provide partial mitigation of this concern.   - First, data were collected via Prolific, a platform that has demonstrated substantially lower rates of careless responding compared with other online recruitment platforms such as Amazon Mechanical Turk, due to its pre-screening procedures, reputation-based participant selection, and financial incentive structures (Peer et al., 2022). - Second, participants who did not complete the ETMCQ-R were excluded from all analyses (n = 10, 1.9%), and the scale's seven-point response format with bidirectional item content provides some implicit sensitivity to random or acquiescent responding patterns. - Third, the observed psychometric properties of the data (including adequate item-level variability across the full response range, expected inter-factor correlations consistent with theoretical predictions, and convergent validity with multiple external criteria) are inconsistent with substantial contamination from random responding, which would attenuate all correlations and distort factor structure rather than producing the theoretically coherent patterns we observed.   We have added an explicit acknowledgement of the absence of formal attention checks to the Limitations section of the revised manuscript, noting that future studies using the ETMCQ-R-6 in online contexts should incorporate instructed-response items to allow formal careless responding screening. We have also added Ward & Meade (2023) and Peer et al. (2022) to the reference list. |
| 6. The manuscript calls the ETMCQ-R-6 a "screener" appropriate for "clinical screening" and "treatment monitoring." Yet the validation was performed entirely on a non-clinical community sample. No clinical group was studied, no sensitivity/specificity analyses were conducted, no clinical cut-off scores were determined, and no test-retest reliability was collected. A "screener" implies diagnostic or prognostic utility, which is wholly unsupported here. All language referring to clinical applications must be removed or drastically tempered. | We agree with the reviewer that the term "screener" carries clinical connotations that are not fully supported by a community validation sample alone, and that individual-level clinical applications require additional evidence that the current study does not provide. We have revised the language throughout the manuscript accordingly. |
| 7. Items were retained through iterative deletion of the lowest-loading indicators from the full 18-item CFA model. No methodological references support this procedure. Modern scale-shortening research demonstrates that stepwise deletion based solely on factor loadings capitalizes on chance, rarely balances multiple psychometric criteria simultaneously, and yields short forms with impaired criterion validity. The state-of-the-art involves optimization algorithms like Ant Colony Optimization or Genetic Algorithms (Schroeders et al., 2016). The authors should acknowledge that their selection method is not best practice and discuss its limitations. | We agree with the reviewer that stepwise CFA-based item deletion does not represent the current state of the art for short-form development, and we thank them for pointing to Schroeders et al. (2016) as the relevant methodological reference. We have added explicit acknowledgement of this limitation to both the Methods and Discussion sections of the revised manuscript.  We wish to clarify, however, that item selection in the present study was not based solely on factor loadings. As described in the Methods, the final item selection applied a joint criterion incorporating factor loading strength, internal consistency contribution, and content coverage – specifically selecting item pairs that represented conceptually complementary rather than redundant facets of each construct. This multi-criterion approach partially mitigates the risk of pure chance capitalization that the reviewer correctly identifies as a weakness of loading-only stepwise deletion.  We also note that the item selection procedure followed the same approach used in the derivation of the parent ETMCQ-R (Campbell et al., 2025), ensuring methodological continuity and comparability between the long and short form.  Nonetheless, we acknowledge that modern metaheuristic approaches such as Ant Colony Optimization or Genetic Algorithms simultaneously optimize across multiple psychometric targets — including fit indices, reliability, and criterion validity — and are inherently less susceptible to sample-specific capitalization than iterative manual deletion and have added this to the discussion of our findings. |
| 8. The manuscript states that items were chosen to represent "complementary facets" to avoid redundancy, but provides no information about how this was evaluated, who conducted it, or how consensus was reached (e.g., formal expert consensus vs. informal author judgment). Content validity in scale shortening requires a transparent, auditable process. Without this detail, claims of adequate content coverage are unverifiable. | We thank the reviewer for identifying this gap in methodological transparency. The reviewer is correct that the original manuscript did not describe the content validity evaluation process in sufficient detail. We have added a description of this process to the Methods section.  The content validity evaluation was conducted by the core author team following the quantitative CFA-based item reduction. For each subscale, the two retained item pairs were evaluated against the theoretical definitions of epistemic trust, mistrust, and credulity as specified in the foundational theoretical framework (Fonagy & Allison, 2014) and operationalized in the parent ETMCQ-R validation study (Campbell et al., 2025). The evaluation assessed whether each retained item pair captured conceptually distinct and complementary facets of the underlying construct rather than representing redundant expressions of the same theme.  For the *Trust subscale*, the two retained items were judged to reflect benevolence appraisal — perceiving others' input as offered with supportive intent — and the perceived personal relevance of socially transmitted information, together capturing the core concept of epistemic openness to guidance from others. For the *Mistrust subscale*, the retained items were judged to represent sensitivity to potential malevolence and protective resistance to influence, reflecting the two primary components of epistemic hypervigilance.  For the *Credulity subscale*, the retained items were judged to capture suggestibility and over-openness to social information, representing complementary aspects of insufficient epistemic filtering.  Consensus was reached through discussion among co-authors with expertise in epistemic trust theory, psychometric methodology, and clinical application, with final item assignments confirmed by the lead and senior authors. No formal inter-rater reliability procedure was employed, which we acknowledge as a limitation of the content validity process. |
| 9. The introduction does not explain why attachment styles, social support, mentalizing, borderline features, and resilience were chosen as criterion measures. Theoretical links between epistemic stances and each of these constructs are never articulated until the methods section. The conceptual rationale for the nomological network belongs in the introduction to motivate the study's hypotheses. | We agree with the reviewer that the theoretical rationale for the criterion measures should appear in the Introduction to motivate the study's hypotheses rather than being relegated to the Methods section. We have added a paragraph to the Introduction that articulates the theoretical basis for each criterion domain selected. The corresponding text in the Methods section has been retained but condensed to avoid repetition. |
| 10. The thresholds for evaluating short-form-to-long-form correlation attenuation (<20% negligible, 20–30% acceptable, >30% problematic) are presented without any citation or empirical rationale. The authors must explain this reasoning and acknowledge the lack of precedent. | We thank the reviewer for this precise observation. The reviewer is correct that the percentage-based attenuation thresholds (<20% negligible, 20–30% acceptable, >30% problematic) were presented without citation or empirical justification. We have therefore removed these thresholds from the manuscript entirely and replaced the evaluation with a descriptive interpretation based on Cohen's q effect sizes, which have an established empirical rationale and published benchmarks (Cohen, 1988). The percentage change values are retained in the tables as descriptive information but are no longer evaluated against arbitrary benchmarks. |
| 11. The cross-sectional mediation models are tangential to the core psychometric aim of short-form validation and distract from the measurement questions. Interpretation as "mediation" is also inappropriate with cross-sectional data; these are indirect effects that cannot support causal inference. I recommend removing the mediation section entirely to sharpen the paper's focus. If retained, the inability to infer causality must be explicitly and prominently stated. | We agree with the reviewer on both points. The mediation section was originally included to mirror the analyses reported in the parent ETMCQ-R validation study (Campbell et al., 2025), but the reviewer is correct that cross-sectional indirect effect analyses are tangential to the core psychometric aim of short-form validation and that causal inference is not supported by the cross-sectional design. We have therefore removed the mediation section entirely.  Instead, we have added incremental predictive validity analyses to address the psychometrically relevant question of whether each subscale contributes unique and non-redundant variance to key outcomes beyond the other subscales. These analyses directly, providing evidence for the discriminant validity of the three-factor structure. Mistrust was entered in Step 1, followed by credulity in Step 2 and trust in Step 3, given the theoretical and empirical evidence linking mistrust most consistently to psychopathology. A sensitivity analysis with reversed entry order was conducted to confirm that incremental contributions were robust to entry sequence. This analytical approach is more directly relevant to short-form validation and avoids the causal inference concerns the reviewer correctly identified. |
| 12. Mistrust shows α = 0.68, ρ = 0.68, and ω = 0.68, all below the conventional 0.70 minimum. For two-item scales, the Spearman-Brown coefficient is more appropriate than Cronbach's alpha (Eisinga et al., 2013), but even that value fails to reach 0.70. The authors must discuss the practical implications of this low reliability for individual-level research and avoid characterizing it as "adequate." | We agree fully with the reviewer. The term "adequate" has been removed wherever it was used to characterize Mistrust reliability. We acknowledge explicitly that all reliability indices for the Mistrust subscale — Cronbach's α, Spearman-Brown ρ, and McDonald's ω — converge at .68, falling below the conventional threshold of .70. As the reviewer correctly notes, the convergence of all three indices on the same value confirms that this represents a genuine reliability limitation rather than a measurement artefact of Cronbach's alpha specifically, and the alpha-underestimation argument cannot be invoked here.  We have revised the relevant text throughout the manuscript to accurately characterize the Mistrust reliability as below the conventional threshold, and have added explicit discussion of the practical implications. Specifically, we note that reliability below .70 limits the precision of individual-level score interpretation and increases measurement error at the item level, meaning that the Mistrust subscale should not be used for high-stakes individual clinical decisions. At the group level, where measurement error averages out across participants, the subscale retains utility for research applications examining associations between epistemic mistrust and other constructs, which is consistent with the acceptable correlations with external validity measures observed in the present study. We also note that the intentionally heterogeneous content of the two Mistrust items — capturing both sensitivity to potential malevolence and protective resistance to influence as complementary rather than redundant facets — partially explains the lower inter-item correlation, representing a deliberate trade-off between content breadth and internal consistency that is inherent to ultra-brief two-item measurement. |
| 13. A measure proposed for treatment monitoring must have demonstrated temporal stability. The absence of test-retest data is a critical omission that should be acknowledged as a major limitation. | We agree fully with the reviewer. Test-retest reliability is an essential psychometric property for any instrument intended to monitor change over time, and its absence is a genuine limitation of the current study. We have addressed this in two ways in the revised manuscript.  First, as noted in our response to Major Comment 6, we have removed the unsupported claim that the ETMCQ-R-6 is validated for treatment monitoring at the individual level. The instrument is now consistently described as suitable for research and population-level assessment, with individual-level treatment monitoring requiring dedicated further validation. This directly resolves the logical tension the reviewer identifies: a measure cannot be recommended for treatment monitoring without test-retest data demonstrating that its scores are stable over time in the absence of intervention and sensitive to change in the presence of intervention.  Second, the absence of test-retest reliability data has been added as an explicit major limitation in the Limitations section, alongside a description of what would be required before the ETMCQ-R-6 could be recommended for treatment monitoring. We note that demonstrating treatment monitoring utility would require not only test-retest reliability but also sensitivity-to-change data, minimally important difference estimates, and ideally validation in a clinical sample undergoing a relevant intervention — none of which are available in the current study. |
| 14. The introduction observes that the parent trust scale has repeatedly shown substantially weaker associations with outcomes than the mistrust and credulity scales. In the present study, trust exhibits negligible correlations with anxiety, depression, borderline personality features, and resilience, a pattern that directly replicates the problematic introduction. Yet the discussion entirely sidesteps this. The authors must address why epistemic trust fails to relate to psychopathology constructs and whether the trust subscale truly contributes incremental validity worthy of retention. | We thank the reviewer for pushing us to engage directly with this pattern. We have added a dedicated paragraph to the Discussion that addresses both questions explicitly.  We agree that epistemic trust shows negligible correlations with psychopathology outcomes, and that the Discussion previously sidestepped this. The incremental validity analyses in the revised manuscript confirm this directly: trust does not contribute significant variance beyond mistrust and credulity for general psychological distress or borderline personality features (Δadj. R² ≤ .005, p ≥ .098 across all entry orders).  However, we argue this reflects construct-level specificity rather than measurement failure. Epistemic trust is theoretically conceptualized as a facilitator of adaptive social learning and interpersonal openness rather than a direct marker of clinical risk (Fonagy & Allison, 2014). Its associations with psychopathology would accordingly be expected to be weak in cross-sectional community samples. The incremental validity analyses support this interpretation: the Trust subscale alone explained 21.9% of variance in attachment avoidance — the largest primary contribution of any subscale across all outcomes examined — while mistrust and credulity contributed negligible additional variance once trust was accounted for. This pattern of outcome-specific dominance is precisely what the epistemic trust framework predicts: trust operates through interpersonal mechanisms linked to attachment and social openness rather than through direct psychopathology pathways.  We therefore retain the Trust subscale while acknowledging its limitations for psychopathology prediction in community samples, and recommend it be interpreted as a measure of adaptive interpersonal openness whose clinical utility is best evaluated in longitudinal treatment studies. Removing it – as suggested by Asgarizadeh et al. (2023) – would eliminate the only component of the ETMCQ-R-6 capturing the adaptive end of the epistemic stance spectrum and would undermine the theoretical differentiation foundational to the Fonagy and Allison (2014) framework. |
| Minor Comments |  |
| 1. The title reads "EMTCQ-R-6"; the correct acronym is "ETMCQ-R-6." | Corrected as suggested. |
| 2. Section 3.4 is labeled "Psychometric evaluation of the ETMCQ-R-6," but the entire results section is a psychometric evaluation. Use a more specific heading. | Corrected as suggested. |
| 3. Criterion measures formatting: All assessment instruments are presented in one dense paragraph. Each measure should be introduced in its own paragraph with systematic information: construct, number of items, subscales, response scale (anchors), and interpretation of scores. | We note that the assessment instruments used in this study are identical to those described in the parent ETMCQ-R validation study (Campbell et al., 2025), where full psychometric details for each measure are reported in the same format the reviewer requests. To avoid extensive redundancy with an already published paper that readers can consult for full instrument descriptions, we have elected to retain a concise format in the present manuscript while ensuring that the key information — construct assessed, number of items, response scale, and score interpretation — is present for each measure. We have restructured the Measures section so that each instrument is described in its own paragraph, providing the systematic information the reviewer requests, while keeping descriptions appropriately brief given the prior publication of full details in Campbell et al. (2025). |
| 4. The RMSEA for the final model has a 95% CI of [0.042–0.106]; the upper bound exceeds 0.10, suggesting that poor fit cannot be ruled out. This should be explicitly noted. | A brief factual note has been added to the Results acknowledging that the RMSEA upper bound exceeds .10. The substantive interpretation and cross-sample contextualisation have been placed in the Discussion, where the finding is explained in terms of sample size sensitivity of RMSEA confidence intervals and contextualised against the German replication RMSEA of .060 [.047, .074]. |
| 5. The rationale for using a 70/30 split rather than a 50/50 split is absent. The precise randomization procedure (simple random? stratified?) must be described | We agree this information was missing. The Methods section has been updated to provide both the rationale and the randomization procedure.  The 70/30 split was chosen to maximise the derivation subsample size while retaining a sufficient holdout subsample for an internal replication check. With a total sample of N = 515, a 50/50 split would have yielded derivation and holdout subsamples of approximately N = 257 each — a derivation sample that would be insufficient for stable CFA estimation of a six-item three-factor model with adequate power. The 70/30 split provides a derivation subsample of N = 360, which better supports stable parameter estimation, while retaining N = 155 for the holdout check. Cases were allocated to subsamples using simple random assignment with a fixed random seed in R, without stratification. We acknowledge that this internal split does not constitute independent validation, as noted in the revised manuscript; the independent German replication sample (N = 2,522) serves as the primary cross-sample validation evidence. |
| 6. Please clarify whether "gender" refers to biological sex assigned at birth or self-identified gender identity. If these are conflated, terminology should be corrected throughout. | We thank the reviewer for raising this important terminological point. Data were collected via Prolific using the platform's standard demographic question, which asks participants to report their gender identity rather than biological sex assigned at birth. The response options presented to participants were female, male, non-binary, and prefer not to answer, consistent with self-identified gender identity rather than biological sex. The term "gender" as used throughout the manuscript therefore refers to self-identified gender identity and the terminology is appropriate. We have added a clarifying sentence to the Methods section making this explicit, and have ensured that the term "gender" is used consistently throughout rather than being conflated with biological sex. |
| 7. When discussing the mediation analyses, the text refers to "ETMCQ-R" subscales; specify that the short-form ETMCQ-R-6 was used. | The mediation section has been removed entirely from the revised manuscript in response to Major Comment 11, and replaced with hierarchical regression analyses examining the incremental predictive validity of the ETMCQ-R-6 subscales. The ETMCQ-R-6 is specified consistently by name throughout the replacement section. |
| 8. Several discussion paragraphs merely restate results without offering interpretation, theoretical integration, or critical reflection. These passages should be condensed. | The Discussion has been revised throughout to reduce result restatement and strengthen interpretive and theoretical content. Specifically, passages that summarised numerical findings already reported in the Results section have been condensed to single sentences or removed entirely, with the freed space used to expand theoretical integration and critical reflection. The revisions to the Discussion required by Major Comments 7, 8, 12, and 14 have simultaneously added substantive interpretive content, meaning the revised Discussion is both leaner in its descriptive passages and richer in its analytical content than the original. |
| 9. There are also several grammar and syntax errors that warrant careful attention. | We thank the reviewer for flagging this. The manuscript has been carefully proofread in its entirety and grammar and syntax errors have been corrected throughout. |

| Reviewer #2: The authors investigate the reliability and validity of a short version of the Epistemic Trust Mistrust and Credulity Questionnaire revised, complementing a line of research on the ETMCQ to improve the scale with regards to psychometric properties and clinical use. The paper is very well written and the study methodologically sound. | We thank Reviewer 2 for the positive assessment of the manuscript and for the constructive suggestions. We address each point below. |
| --- | --- |
| I have a few comments which I recommend addressing:  • Reliability of mistrust scale is not really adequate, which should be mentioned (and corrected) in the abstract | The abstract has been revised to accurately distinguish reliability levels across subscales. The previous characterisation of all subscales as showing acceptable reliability has been replaced with an accurate summary noting that trust and credulity demonstrated acceptable reliability while the Mistrust subscale fell below the conventional threshold. |
| • Calculate also dynamic cutoffs based on the data at hand | We have calculated percentile-based cutoffs from the UK sample data and added these as a supplementary table. Specifically, we report the 25th, 50th, and 75th percentile scores for each subscale, stratified by gender and age group, as empirically derived reference points complementing the normative means and standard deviations already reported. We note in the supplementary table that these cutoffs are derived from a quota-balanced community sample and should be interpreted as descriptive reference values rather than clinical diagnostic thresholds, pending dedicated clinical validation. |
| • The following section in the discussion should me rewritten as to better express the point also for readers who are not as much informed about the history of the ETMCQ: Interestingly, the six items of the ETMCQ-R stem from the first version of the ETMCQ which would allow the validation of the short form in previously collected data sets for the validation of the initial ETMCQ form. Importantly, the short form showed high correlations and excellent congruence with the 18-item ETMCQ-R, indicating that the abbreviated version closely aligns with the essential psychometric and conceptual properties of the full instrument… | We agree that the original passage assumed prior familiarity with the instrument's development history that many readers will not have. The paragraph has been rewritten for accessibility. |
| • Regarding this argument: It has been suggested, that Cronbach Alpha frequently underestimated the true reliability and should be interpreted with caution..  what about the other reliability measures provided? | This is an important clarifying point that we have addressed directly in the revised Discussion. The argument that alpha underestimates reliability applies to specific scale structures — particularly those with heterogeneous item content or non-tau-equivalent items — and cannot be invoked uniformly. In the present case, the Spearman-Brown coefficient, McDonald's ω, and the inter-item correlation all converge on values very similar to alpha across all three subscales. For the Mistrust subscale in particular, all indices converge at .68, confirming that the below-threshold reliability is a genuine finding rather than an alpha-specific artefact. We have removed the alpha-underestimation argument from the manuscript and replaced it with an accurate statement that the convergence of all reliability indices confirms the genuine nature of the Mistrust subscale's reliability limitation. For Trust and Credulity, the convergence of all indices at or above .75 similarly confirms that their acceptable reliability is not inflated by any index-specific artefact. |
| Formal:  • MZQ literature is missing | MZQ reference added to the reference list and cited at first mention in the Measures section. |
| • Information on analysis software is missing | Software information has been added to the Statistical Analysis section. |
| • introduce CFA abbreviation at first mention of confirmatory factor analysis | The abbreviation CFA is now introduced at its first occurrence in the text as "confirmatory factor analysis (CFA)" and used consistently thereafter. |
