# Supplementary material for: Can we trust this short scale? Validation of a Brief Self-Report Measure of Epistemic Trust, Mistrust and Credulity (ETMCQ-R-6) in a sample of the U.K. general population
Source: PLOS Ment Health. 2026 Aug 31;3(8):e0000591. doi: 10.1371/journal.pmen.0000591 (PMC13529005; doi:10.1371/journal.pmen.0000591)
Supplement: Peer review history

# Peer review: Decision Letter 0

**DOI:** 10.1371/journal.pmen.0000591.r001

**Authors:** Katrien Janin

20 May 2026

PMEN-D-26-00154

Can we trust this short scale? Validation of the Epistemic Trust, Mistrust and Credulity Screener (EMTCQ-R-6) in a sample of the U.K. general population

PLOS Mental Health

Dear Dr. Riedl,

Thank you for submitting your manuscript to PLOS Mental Health. After careful consideration, we feel that it has merit but does not fully meet PLOS Mental Health’s publication criteria as it currently stands. Therefore, we invite you to submit a revised version of the manuscript that addresses the points raised during the review process.

Your manuscript has been evaluated by two reviewers. While they indicate interest in your work, they also identified several concerns that need to be addressed. Please consider their comments carefully when preparing your revised manuscript and your point‑by‑point response.

Please submit your revised manuscript by Jul 18 2026 11:59PM. If you will need more time than this to complete your revisions, please reply to this message or contact the journal office at mentalhealth@plos.org. When you're ready to submit your revision, log on to https://www.editorialmanager.com/pmen/ and select the 'Submissions Needing Revision' folder to locate your manuscript file.

Please include the following items when submitting your revised manuscript:

- A letter that responds to each point raised by the editor and reviewer(s). You should upload this letter as a separate file labeled 'Response to Reviewers'.
- A marked-up copy of your manuscript that highlights changes made to the original version. You should upload this as a separate file labeled 'Revised Manuscript with Track Changes'.
- An unmarked version of your revised paper without tracked changes. You should upload this as a separate file labeled 'Manuscript'.

Guidelines for resubmitting your figure files are available below the reviewer comments at the end of this letter.

As the corresponding author, your ORCID iD is verified in the submission system and will appear in the published article. PLOS supports the use of ORCID, and we encourage all coauthors to register for an ORCID iD and use it as well. Please encourage your coauthors to verify their ORCID iD within the submission system before final acceptance, as unverified ORCID iDs will not appear in the published article. *Only*  the individual author can complete the verification step; PLOS staff *cannot*  verify ORCID iDs on behalf of authors.

We look forward to receiving your revised manuscript.

Kind regards,

Katrien G. Janin, PhD

Staff Editor

PLOS Mental Health

**Journal Requirements:**

1. Please amend your detailed Financial Disclosure statement. This is published with the article. It must therefore be completed in full sentences and contain the exact wording you wish to be published.

i. Please clarify all sources of financial support for your study. List the grants, grant numbers, and organizations that funded your study, including funding received from your institution. Please note that suppliers of material support, including research materials, should be recognized in the Acknowledgements section rather than in the Financial Disclosure.

ii. State the initials, alongside each funding source, of each author to receive each grant. For example: "This work was supported by the National Institutes of Health (####### to AM; ###### to CJ) and the National Science Foundation (###### to AM)."

iii. State what role the funders took in the study. If the funders had no role in your study, please state: “The funders had no role in study design, data collection and analysis, decision to publish, or preparation of the manuscript.”

iv. If any authors received a salary from any of your funders, please state which authors and which funders.

2. In the online submission form, you indicated that “The data that support the findings of this study are available from the corresponding author, D.R. upon reasonable request”.

All PLOS journals now require all data underlying the findings described in their manuscript to be freely available to other researchers, either

1. In a public repository,

2. Within the manuscript itself, or

3. Uploaded as supplementary information.

This policy applies to all data except where public deposition would breach compliance with the protocol approved by your research ethics board. If your data cannot be made publicly available for ethical or legal reasons (e.g., public availability would compromise patient privacy), please explain your reasons by return email and your exemption request will be escalated to the editor for approval. Your exemption request will be handled independently and will not hold up the peer review process, but will need to be resolved should your manuscript be accepted for publication. One of the Editorial team will then be in touch if there are any issues.

3. Please upload separate figure files in .tif or .eps format. Also, remove the figures from your manuscript file but keep the legends.

For more information about figure files please see our guidelines:

https://journals.plos.org/mentalhealth/s/figures

https://journals.plos.org/mentalhealth/s/figures#loc-file-requirements

4. We have noticed that you have uploaded Supporting Information files, but you have not included a list of legends. Please add a full list of legends for your Supporting Information files after the references list.

If the reviewer comments include a recommendation to cite specific previously published works, please review and evaluate these publications to determine whether they are relevant and should be cited. There is no requirement to cite these works unless the editor has indicated otherwise.

**Additional Editor Comments (if provided):**

[Note: HTML markup is below. Please do not edit.]

Reviewers' comments:

Reviewer's Responses to Questions

**Comments to the Author**

1. Does this manuscript meet PLOS Mental Health’s publication criteria?

Reviewer #1: Partly

Reviewer #2: Yes

**********

2. Has the statistical analysis been performed appropriately and rigorously?

Reviewer #1: No

Reviewer #2: Yes

**********

3. Have the authors made all data underlying the findings in their manuscript fully available (please refer to the Data Availability Statement at the start of the manuscript PDF file)?

The PLOS Data policy

Reviewer #1: No

Reviewer #2: No

**********

4. Is the manuscript presented in an intelligible fashion and written in standard English?

Reviewer #1: Yes

Reviewer #2: Yes

**********

**Reviewer #1:** This manuscript describes the development of the ETMCQ-R-6, a six-item short form of the Revised Epistemic Trust, Mistrust and Credulity Questionnaire, derived from the full 18-item ETMCQ-R using a UK quota-balanced community sample (N = 515). The authors selected two items per subscale based on CFA, internal consistency, and content coverage. They report acceptable model fit, adequate reliability for trust and credulity but marginal reliability for mistrust (α = 0.68), configural/metric/structural invariance across gender, and mediation patterns consistent with prior work. The paper concludes that the ETMCQ-R-6 is a psychometrically stable brief screener for research and clinical use.

My recommendation is Major Revisions. The manuscript suffers from two foundational flaws that cannot be resolved within the current framework merely by disclosing them as limitations. First, the short form is developed and tested on the identical sample used for the parent ETMCQ-R validation, which maximally capitalizes on sample-specific variance and artificially inflates apparent psychometric correspondence. Second, the decision to construct each factor from only two items directly transgresses the widely accepted three-indicator rule for latent variable models (Hair et al., 2019), producing an inadmissible Heywood case (β = 1.09 for a trust item) and a second borderline Heywood case in the holdout sample. Because of these structural limitations, the paper does not provide credible evidence that the ETMCQ-R-6 functions as a valid short form. I believe a revision is only acceptable if the authors (a) reconceptualize the short form as a nine-item instrument (i.e., at least three items per subscale) to satisfy basic identification requirements, and (b) validate this new short form with at least some new, independent data that were not used in the development of the parent ETMCQ-R. The detailed comments below elaborate on these and additional issues that must be addressed.

Major Comments

1. The manuscript states that the data were collected between March 16 and March 23, 2023, from 525 adults via Prolific. This is the exact same dataset that supported the validation of the parent 18-item ETMCQ-R instrument. Deriving a short form and conducting all psychometric analyses on the very same dataset used to validate the parent instrument is a critical methodological error. Smith et al. (2000), in their authoritative paper on short-form development, explicitly warn against this practice, as it capitalizes on sample-specific covariance and leads to overoptimistic estimates of factor loadings, model fit, and short-to-long-form correspondence. The internal 70/30 holdout split does not remedy this, as the data source remains identical. Disclosing this as a limitation is insufficient for publication in a top-tier journal. The authors must commit to validating the short form using a genuinely independent sample.

2. The standardized loading of 1.09 for Trust Item #1 is a Heywood case and an inadmissible solution. This is not a minor problem that can be explained by noting the item is "highly saturated"; it is a structural inevitability of specifying a factor with only two indicators. The three-indicator rule (Hair et al., 2019) explicitly warns that two-indicator factors are locally under-identified and highly fragile. The authors themselves report that the latent construct reliability (H) for trust reached 1.0; a direct mathematical artifact of the Heywood case that cannot be interpreted as perfect reliability. Furthermore, in the 30% holdout sample, a mistrust loading reached 1.00 (β = 0.51–1.00), proving that another two-item factor became unstable. These findings unequivocally show that the six-item, two-indicators-per-factor model is psychometrically unsound. While constraining the trust loadings eliminates the Heywood case computationally, the underlying structural flaw remains: two-item factors fail to provide adequate construct coverage and produce unreliable parameter estimates. In my view, the authors must abandon the current six-item version and develop a nine-item short form (i.e., at least three items per subscale) to achieve proper identification and genuine content sampling.

3. The authors used maximum likelihood (ML) estimation with bootstrapped confidence intervals for factor loadings but retained standard ML for the model χ², CFI, TLI, RMSEA, and SRMR. Given that the items are ordinal (7-point Likert scales) and potential non-normality was acknowledged, this approach is inadequate. Bootstrapping loadings does not correct the model-level test statistic, nor does it provide robust standard errors for all parameters. The appropriate estimators for ordinal indicators are WLSMV (which makes no distributional assumptions and uses polychoric correlations) or MLR. All CFA and invariance analyses must be re-estimated with a robust estimator, and the corresponding adjusted fit indices must be reported.

4. The invariance section contains multiple errors and omissions:

• The framework is incorrectly described: constraints are cumulative. Metric invariance constrains structure and loadings; scalar constrains structure, loadings, and intercepts; etc. The manuscript implies each step operates independently.

• The scalar invariance step is mentioned, but its results (including ΔCFI and Δχ²) are completely absent from the text.

• The abstract and methods claim that invariance across age groups was tested, yet no age-invariance results appear anywhere in the results section.

• The invariance criteria are incomplete: the authors mention ΔCFI ≤ .010 (Cheung & Rensvold, 2002) but omit the corresponding ΔRMSEA guideline (e.g., ΔRMSEA < .015; Chen, 2007).

• Best practice requires testing the CFA model separately within each group (e.g., females and males) to confirm adequate baseline fit before proceeding to multi-group configural invariance.

• A comprehensive table is required, listing χ², df, CFI, RMSEA, and the Δχ², ΔCFI, and ΔRMSEA for each model step.

5. Data were collected online via Prolific, yet the manuscript says nothing about attention checks, instructed-response items, or mechanisms for identifying careless respondents. Given the well-documented threat of inattentive responding in unsupervised web-based samples (Ward & Meade, 2023), this is a serious omission. The lack of screening may have artificially inflated reliability estimates and distorted CFA results. This must be detailed if employed, or heavily acknowledged as a major threat to data quality.

6. The manuscript calls the ETMCQ-R-6 a "screener" appropriate for "clinical screening" and "treatment monitoring." Yet the validation was performed entirely on a non-clinical community sample. No clinical group was studied, no sensitivity/specificity analyses were conducted, no clinical cut-off scores were determined, and no test-retest reliability was collected. A "screener" implies diagnostic or prognostic utility, which is wholly unsupported here. All language referring to clinical applications must be removed or drastically tempered.

7. Items were retained through iterative deletion of the lowest-loading indicators from the full 18-item CFA model. No methodological references support this procedure. Modern scale-shortening research demonstrates that stepwise deletion based solely on factor loadings capitalizes on chance, rarely balances multiple psychometric criteria simultaneously, and yields short forms with impaired criterion validity. The state-of-the-art involves optimization algorithms like Ant Colony Optimization or Genetic Algorithms (Schroeders et al., 2016). The authors should acknowledge that their selection method is not best practice and discuss its limitations.

8. The manuscript states that items were chosen to represent "complementary facets" to avoid redundancy, but provides no information about how this was evaluated, who conducted it, or how consensus was reached (e.g., formal expert consensus vs. informal author judgment). Content validity in scale shortening requires a transparent, auditable process. Without this detail, claims of adequate content coverage are unverifiable.

9. The introduction does not explain why attachment styles, social support, mentalizing, borderline features, and resilience were chosen as criterion measures. Theoretical links between epistemic stances and each of these constructs are never articulated until the methods section. The conceptual rationale for the nomological network belongs in the introduction to motivate the study's hypotheses.

10. The thresholds for evaluating short-form-to-long-form correlation attenuation (<20% negligible, 20–30% acceptable, >30% problematic) are presented without any citation or empirical rationale. The authors must explain this reasoning and acknowledge the lack of precedent.

11. The cross-sectional mediation models are tangential to the core psychometric aim of short-form validation and distract from the measurement questions. Interpretation as "mediation" is also inappropriate with cross-sectional data; these are indirect effects that cannot support causal inference. I recommend removing the mediation section entirely to sharpen the paper's focus. If retained, the inability to infer causality must be explicitly and prominently stated.

12. Mistrust shows α = 0.68, ρ = 0.68, and ω = 0.68, all below the conventional 0.70 minimum. For two-item scales, the Spearman-Brown coefficient is more appropriate than Cronbach's alpha (Eisinga et al., 2013), but even that value fails to reach 0.70. The authors must discuss the practical implications of this low reliability for individual-level research and avoid characterizing it as "adequate."

13. A measure proposed for treatment monitoring must have demonstrated temporal stability. The absence of test-retest data is a critical omission that should be acknowledged as a major limitation.

14. The introduction observes that the parent trust scale has repeatedly shown substantially weaker associations with outcomes than the mistrust and credulity scales. In the present study, trust exhibits negligible correlations with anxiety, depression, borderline personality features, and resilience, a pattern that directly replicates the problematic introduction. Yet the discussion entirely sidesteps this. The authors must address why epistemic trust fails to relate to psychopathology constructs and whether the trust subscale truly contributes incremental validity worthy of retention.

Minor Comments

1. The title reads "EMTCQ-R-6"; the correct acronym is "ETMCQ-R-6."

2. Section 3.4 is labeled "Psychometric evaluation of the ETMCQ-R-6," but the entire results section is a psychometric evaluation. Use a more specific heading.

3. Criterion measures formatting: All assessment instruments are presented in one dense paragraph. Each measure should be introduced in its own paragraph with systematic information: construct, number of items, subscales, response scale (anchors), and interpretation of scores.

4. The RMSEA for the final model has a 95% CI of [0.042–0.106]; the upper bound exceeds 0.10, suggesting that poor fit cannot be ruled out. This should be explicitly noted.

5. The rationale for using a 70/30 split rather than a 50/50 split is absent. The precise randomization procedure (simple random? stratified?) must be described.

6. Please clarify whether "gender" refers to biological sex assigned at birth or self-identified gender identity. If these are conflated, terminology should be corrected throughout.

7. When discussing the mediation analyses, the text refers to "ETMCQ-R" subscales; specify that the short-form ETMCQ-R-6 was used.

8. Several discussion paragraphs merely restate results without offering interpretation, theoretical integration, or critical reflection. These passages should be condensed.

9. There are also several grammar and syntax errors that warrant careful attention.

**Reviewer #2:** The authors investigate the reliability and validity of a short version of the Epistemic Trust Mistrust and Credulity Questionnaire revised, complementing a line of research on the ETMCQ to improve the scale with regards to psychometric properties and clinical use.

The paper is very well written and the study methodologically sound.

I have a few comments which I recommend addressing:

• Reliability of mistrust scale is not really adequate, which should be mentioned (and corrected) in the abstract

• Calculate also dynamic cutoffs based on the data at hand

• The following section in the discussion should me rewritten as to better express the point also for readers who are not as much informed about the history of the ETMCQ: Interestingly, the six items of the ETMCQ-R stem from the first version of the ETMCQ which would allow the validation of the short form in previously collected data sets for the validation of the initial ETMCQ form. Importantly, the short form showed high correlations and excellent congruence with the 18-item ETMCQ-R, indicating that the abbreviated version closely aligns with the essential psychometric and conceptual properties of the full instrument…

• Regarding this argument: It has been suggested, that Cronbach Alpha frequently underestimated the true reliability and should be interpreted with caution.. � what about the other reliability measures provided?

Formal:

• MZQ literature is missing

• Information on analysis software is missing

• introduce CFA abbreviation at first mention of confirmatory factor analysis

**********

what does this mean?). If published, this will include your full peer review and any attached files.

**Do you want your identity to be public for this peer review?**  If you choose “no”, your identity will remain anonymous but your review may still be made public.

For information about this choice, including consent withdrawal, please see our Privacy Policy

Reviewer #1: No

Reviewer #2: No

**********

[NOTE: If reviewer comments were submitted as an attachment file, they will be attached to this email and accessible via the submission site. Please log into your account, locate the manuscript record, and check for the action link "View Attachments". If this link does not appear, there are no attachment files.]

**Figure Resubmissions:**

After uploading your figures to PLOS’s NAAS tool - https://ngplosjournals.pagemajik.ai/artanalysis, NAAS will process the files provided and display the results in the "Uploaded Files" section of the page as the processing is complete. If the uploaded figures meet our requirements (or NAAS is able to fix the files to meet our requirements), the figure will be marked as "fixed" above. If NAAS is unable to fix the files, a red "failed" label will appear above. When NAAS has confirmed that the figure files meet our requirements, please download the file via the download option, and include these NAAS processed figure files when submitting your revised manuscript.

# Author comment: Author response to Decision Letter 1

**DOI:** 10.1371/journal.pmen.0000591.r002

26 Jun 2026

Attachment Submitted filename: PTP Response.docx

# Peer review: Decision Letter 1

**DOI:** 10.1371/journal.pmen.0000591.r003

**Authors:** Emanuele Maria Merlo

21 Jul 2026

Can we trust this short scale? Validation of the Epistemic Trust, Mistrust and Credulity Screener (EMTCQ-R-6) in a sample of the U.K. general population

PMEN-D-26-00154R1

Dear Dr. Riedl,

We are pleased to inform you that your manuscript 'Can we trust this short scale? Validation of the Epistemic Trust, Mistrust and Credulity Screener (EMTCQ-R-6) in a sample of the U.K. general population' has been provisionally accepted for publication in PLOS Mental Health.

Before your manuscript can be formally accepted you will need to complete some formatting changes, which you will receive in a follow up email. A member of our team will be in touch with a set of requests.

Please note that your manuscript will not be scheduled for publication until you have made the required changes, so a swift response is appreciated.

IMPORTANT: The editorial review process is now complete. PLOS will only permit corrections to spelling, formatting or significant scientific errors from this point onwards. Requests for major changes, or any which affect the scientific understanding of your work, will cause delays to the publication date of your manuscript.

If your institution or institutions have a press office, please notify them about your upcoming paper to help maximize its impact. If they'll be preparing press materials, please inform our press team as soon as possible -- no later than 48 hours after receiving the formal acceptance. Your manuscript will remain under strict press embargo until 2 pm Eastern Time on the date of publication. For more information, please contact mentalhealth@plos.org.

Thank you again for supporting Open Access publishing; we are looking forward to publishing your work in PLOS Mental Health.

Best regards,

Emanuele Maria Merlo, Ph.D

Academic Editor

PLOS Mental Health

***********************************************************

Dear Professor Riedl,

I have now carefully evaluated the revised version of your manuscript together with your detailed responses to the reviewers' comments.

I appreciate the considerable effort invested in the revision process and the thorough manner in which the reviewers' observations have been addressed. The revised manuscript has been substantially strengthened, both in terms of methodological rigour and overall clarity, and I believe it now represents a valuable contribution to the literature on epistemic trust and mental health.

Accordingly, I have recommended to the Journal that your manuscript be accepted for publication.

I wish you and your co-authors every success with your research.

Best regards

Emanuele Maria Merlo

University of Messina, Messina, Italy

Reviewer Comments (if any, and for reference):

Reviewer's Responses to Questions

**Comments to the Author**

Reviewer #1: All comments have been addressed

**********

publication criteria?

Reviewer #1: Yes

**********

3. Has the statistical analysis been performed appropriately and rigorously?

Reviewer #1: Yes

**********

4. Have the authors made all data underlying the findings in their manuscript fully available (please refer to the Data Availability Statement at the start of the manuscript PDF file)?

The PLOS Data policy

Reviewer #1: Yes

**********

5. Is the manuscript presented in an intelligible fashion and written in standard English?

Reviewer #1: Yes

**********

Reviewer #1: (No Response)

**********

what does this mean?). If published, this will include your full peer review and any attached files.

**Do you want your identity to be public for this peer review?**  If you choose “no”, your identity will remain anonymous but your review may still be made public.

For information about this choice, including consent withdrawal, please see our Privacy Policy

Reviewer #1: No

**********
